# Slow Magnetic Relaxation in {[CoCxAPy)] 2.15 H_2_O}_n_ MOF Built from Ladder-Structured 2D Layers with Dimeric SMM Rungs

**DOI:** 10.3390/molecules26185626

**Published:** 2021-09-16

**Authors:** Ana Arauzo, Elena Bartolomé, Javier Luzón, Pablo J. Alonso, Angelica Vlad, Maria Cazacu, Mirela F. Zaltariov, Sergiu Shova, Juan Bartolomé, Constantin Turta

**Affiliations:** 1 Instituto de Nanociencia y Materiales de Aragón (INMA), CSIC-Universidad de Zaragoza, Pedro Cerbuna 12, 50009 Zaragoza, Spain; jluzon@unizar.es (J.L.); alonso@unizar.es (P.J.A.); barto@unizar.es (J.B.); 2 Department of Mechanical Engineering, Escola Universitària Salesiana de Sarrià (EUSS), Passeig de Sant Joan Bosco, 74, 08017 Barcelona, Spain; ebartolome@euss.es; 3 Centro Universitario de la Defensa, Ctra. de Huesca s/n, 50090 Zaragoza, Spain; 4 Department of Inorganic Polymers, “Petru Poni” Institute of Macromolecular Chemistry, Aleea Gr. Ghica Voda 41A, 700487 Iasi, Romania; avlad@icmpp.ro (A.V.); mcazacu@icmpp.ro (M.C.); zaltariov.mirela@icmpp.ro (M.F.Z.); shova@icmpp.ro (S.S.); 5 Institute of Chemistry, Academy of Sciences of Moldova, Academiei 3, MD-2028 Chisinau, Moldova

**Keywords:** MOF, single molecule magnet, slow magnetic relaxation, 2D coordination polymer, anisotropic exchange, magnetic dimer, Co(II) dimer

## Abstract

We present the magnetic properties of the metal-organic framework {[CoCxAPy]·2.15 H_2_O}_n_ (Cx = bis(carboxypropyl)tetramethyldisiloxane; APy = 4,4′-azopyridine) (**1**) that builds up from the stacking of 2D coordination polymers. The 2D-coordination polymer in the *bc* plane is formed by the adjacent bonding of [CoCxAPy] 1D two-leg ladders with Co dimer rungs, running parallel to the *c*-axis. The crystal packing of 2D layers shows the presence of infinite channels running along the *c* crystallographic axis, which accommodate the disordered solvate molecules. The Co(II) is six-coordinated in a distorted octahedral geometry, where the equatorial plane is occupied by four carboxylate oxygen atoms. Two nitrogen atoms from APy ligands are coordinated in apical positions. The single-ion magnetic anisotropy has been determined by low temperature EPR and magnetization measurements on an isostructural compound {[Zn_0.8_Co_0.2_CxAPy]·1.5 CH_3_OH}_n_ (**2**). The results show that the Co(II) ion has orthorhombic anisotropy with the hard-axis direction in the C_2V_ main axis, lying the easy axis in the distorted octahedron equatorial plane, as predicted by the *ab initio* calculations of the ***g***-tensor. Magnetic and heat capacity properties at very low temperatures are rationalized within a S* = 1/2 magnetic dimer model with anisotropic antiferromagnetic interaction. The magnetic dimer exhibits slow relaxation of the magnetization (SMM) below 6 K in applied field, with a τ_lf_ ≈ 2 s direct process at low frequencies, and an Orbach process at higher frequencies with *U*/k_B_ = 6.7 ± 0.5 K. This compound represents a singular SMM MOF built-up of Co-dimers with an anisotropic exchange interaction.

## 1. Introduction

Metal-organic frameworks (MOFs) are being intensively investigated due to their potential application in areas such as gas storage (e.g., fuel gases like hydrogen [1] and methane), capture of gases (e.g., greenhouse gases) [2], molecular sensing [3], separation [4] and catalysis [5], among others. A huge assortment of MOF topologies and different architectures can be accomplished by chemical design. The common characteristic of these open frameworks is that they are constructed from the assembly of inorganic sub-units (a single metal center, a cluster, or a chain) and organic linkers of different type (carboxylates, phosphonates, azolates, etc.). The high absorption properties of porous MOFs are widely known, but in addition, different functionalities (magnetic [6], electrical [7], or optical [8]) can be incorporated into MOFs by adequately choosing the functional nodes, the organic linkers and their interconnection, or by including functional molecules in the pores.

The study of 3D MOFs counts with an impressive number of publications. The enormous interest in these materials is based on their rigidity and high porosity, which derives in interesting properties and potential applications [9]. On the other hand, the large horizontal and ultra-thin dimensions give the 2D MOF materials very high values for specific surface, atomic surface ratio, and number of active places exposed to surface. These could enhance some capabilities as compared to other nanomaterials or their bulky counterparts and create the premises for developing a new class of materials [10].

Coordination networks with magnetic properties are of particular interest. Four major types of magnetic MOFs are being investigated [11]: (i) MOFs with magnetic cooperativity, (ii) spin-crossover MOFs, (iii) MOFs with magnetocaloric effect (where the nodes possess an isotropic spin), and (iv) MOFs with slow magnetic relaxation, where the nodes are either single-ion magnets (SIMs) or single-molecule magnets (SMMs), or which embed single-chain magnets (SCMs).

Molecular magnets are a class of materials presenting intriguing physics, like slow relaxation and quantum tunneling of the magnetization, with attractive potential applications, e.g., in high density information storage, spintronics, and quantum computing. The controlled organization of nanomagnets into wider molecular edifices offers new exciting possibilities of architectures. On one hand, SMM-MOFs represent ideal models to investigate magneto-structural properties, such as the occurrence of relaxation behavior under different coupling schemes between the magnetic sub-units, or the competition with magnetic ordering, in different types of architectures. From a technological point of view, SMM-MOFs are interesting for quantum computing application, as the framework structure provides spatial separation between the qubit units, minimizing dipolar interactions, which produce the decrease of quantum coherence [11]. Furthermore, functional molecular species may be incorporated into the pores to tune the SMM properties or introduce a second property into the MOF. After the first SMM-MOFs prepared using Mn_4_ clusters [12], many other examples have been reported, based either on lanthanoids [13] or on transition metals [14]. 

Complexes with cobalt ions are particularly good candidates among the 3d transition metals for generating molecules that show slow relaxation of magnetization. Co(II) in an octahedral ligand field is especially interesting due to degenerate t_2g_ levels that are partially occupied, and thus orbital angular momentum is not totally quenched [15]. Early examples of Co(II) SMMs were based on tetranuclear cubanes [16]. Ever since, Co nanomagnets of various dimensionality, SIMs [17], SMM clusters [18], 1D [19], 2D [3] coordination polymers, as well as SCMs assembled in MOFs [20] and a few 3D networks [21] have been reported. Most of them require the application of an external field to show slow magnetic relaxations but there are also some examples of zero-field SMM behavior [16]. 

Examples of coordination polymers (CP’s) containing Co_2_ dimers [15] with either antiferromagnetic (AF) [22] or ferromagnetic (FM) [23] intradimer coupling have been reported, though most works describe only the static magnetic characterization. However, SMM behavior has been demonstrated in a few 2D [24] and 3D [25] CPs built up from Co_2_ units.

The type and geometry of the chemical linkers between the sub-units play a crucial role in determining the architecture and thus the properties of the MOF. Among the numerous ligands, the most employed for the development of cobalt MOFs have been those having oxygen, water (hydroxide, alkoxide, alcohol, and carboxylate) or nitrogen (amine, pyridine, azide, and azole) coordinating atoms, and, in a few cases, sulfur (thiolate) [15]. The developments of metal-containing polymers have further expanded to include silicon: the so called organometallosiloxanes and polymeric derivatives (polyorganometallosiloxanes) containing Si-O-M bonds, or in which the metal is complexed through organic groups attached to the siloxane moiety [26,27]. The metal can be inserted in the main chain or in side position [28]. In previous works, we have demonstrated the success of using highly flexible siloxane bridges in creating Co, Zn, and Mn-based 2D-MOFs [27,29,30]. 

In this work, we have prepared a new MOF that builds up from the staking of 2D CPs, {[CoCxAPy]·2.15 H_2_O}_n_ (**1**), Cx = ditopic chelating organosiloxane ligand (1,3-bis(carboxypropyl)tetramethyldisiloxane dianion) and APy = 4,4′-azopyridine as co-ligands using similar procedure. The 2D-CP is built up by the interconnection of [CoCxAPy] 1D two-leg ladders with Co(II) dimer rungs. We report the magnetic properties of the compound **1**, showing that at low temperatures the dimer magnetism is dominant, where Co-Co rungs are weak AF coupled units exhibiting in-field SMM behavior. The single-ion properties were determined in a heteronuclear analogue, {[Zn_0.80_Co_0.2_CxAPy)]·1.5 CH_3_OH}_n_ (**2**), prepared by partial replacement of Co with non-magnetic Zn.

## 2. Results

### 2.1. Synthesis

Complex **1** was obtained by treating metal perchlorate (Co(ClO_4_)_2_) with a mixture of ligands, 4,4’-azopyridine (APy), and 1,3-bis(carboxy)propyltetramethyldisiloxane (H_2_Cx), in a 2:1:1 molar ratio, in solution in the presence of 2,6-dimethylpyridine as a deprotonating agent. The first evidence of the complexation consists in the spectral changes of the compound (Figure S1a) with respect to the starting compounds: the disappearance of the absorption band characteristic of the carboxyl group from 1715 cm^−1^ and the appearance of the pairs of bands from 1601, 1447, and 1555, 1385 cm^−1^ assigned to ν_as_(COO-) and ν_s_(COO-) vibration bands in carboxylate having both bidentate chelating and bidentate bridging coordination modes [31]. The absorption band corresponding to the -N = N- bond appears at 1425 cm^−1^, displaced from 1410 cm^−1^ in the free 4,4′-azopyridine. The presence of tetramethyldisiloxane fragments is mainly evidenced by the bands from 1047 cm^−1^ (Si-O-Si), 1254, and 839 cm^−1^ (Si-CH_3_). 

The Co-Zn complex **2** has been prepared by the same procedure as complex **1**, by replacing the Co(II) salt with a mixture Co/Zn salts in a molar ratio of 5:95. The IR spectrum (Figure S1b) revealed the characteristic stretches assigned to carboxylate groups (ν_as_ and ν_s_) at about the same wavenumbers as in complex **1**, their magnitude of separation (ν_as_−ν_s_) corresponding to the same coordination mode. The -N = N- stretching vibration is redshifted by 13 cm^−1^ compared to 4,4′-azopyridine, while the Si-O-Si and Si-CH_3_ vibrations are found in the same position as in complex **1**, proving their structural similarity.

The values of the elemental analysis fit well with those calculated for the compound **1**. Slight deviation of the carbon content from the theoretical one in compound **2** could be attributed to some variations in the nature and weight of the crystallization solvents, compared to those found by crystallographic analysis (methanol in this case) as a result of exchange with the environment during the sample manipulation and storage in lab conditions. Calculations based on experimental values would indicate the replacement of most of the crystallization methanol with water, according to formulae {[Zn_0.80_Co_0.2_CxAPy)]·0.5CH_3_OH·1.4H_2_O. This is also supported by the results of the thermogravimetric analysis (Figure S2, Table S1), which show that in the first two stages, the compound **1** loses 5.5 wt% up to 197.9 °C corresponding to cca. 1.6 H_2_O, while the compound **2** loses 8.03 wt% up to 159.6 °C corresponding to 0.5 CH_3_OH and 1.4 H_2_O. The *T*_peak_ values for the main mass losses are 237.5 and 204.2 °C for the compounds **1** and **2**, respectively. Co:Zn molar ratio values estimated by EDX (Figure S3) and EDXRF (Figure S4) are close to each other and to that estimated by crystallographic analysis. 

The DSC trace for the compound **1** (Figure S5) reveals the occurrence of a glass transition (*T*_g_), around 17 °C. This is attributed to the tetramethyldisiloxane spacer in Cx ligand, which is very flexible and unable to develop significant intermolecular interactions, constituting the amorphous part of the structure. This may be a cause for which the diffractogram recorded at room temperature (Figure S6a) is slightly different from that simulated after single crystal X-ray diffraction experiments at 200 K. At room temperature, which is above *T*_g_, the flexible segments begins to relax, causing the interplanary distance to increase [32,33]. Another cause of the non-conformity of the experimental diffractograms with the theoretical ones found in the case of both compounds (Figure S6a,b) could be the adoption by the tetramethyldisiloxane segment of different conformations during the crystallization process that leads to various polymorph modifications. At the same time, the flexibility of the spacer doesn’t affect the chemical model that was used to address the magnetic properties, since the chemical bonds, the coordination function of the ligands, and the coordination environment of the metal atoms do not differ.

### 2.2. Structural Characterization

**Figure 1:**
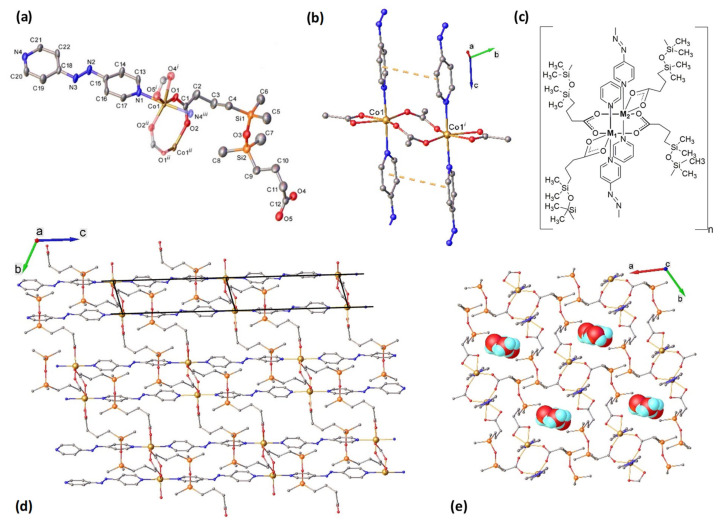
Structure of compound {[CoCxAPy)]·2.15 H_2_O}_n_ (**1**). (**a**) View of the asymmetric unit with atom labelling and thermal ellipsoids at 50% level. H atoms are omitted for clarity. Atoms obtained by symmetry-transformations are displayed semi-transparent. Symmetry codes: ^i)^ *x*, *y* − 1, *z* − 1; ^i)^ 1 − *x*, 1 − *y*, 1 − *z*; ^i)^ *x*, *y*, 1 + *z*. (**b**) Co-Co dimeric unit conforming the ladder rungs. Centroid–to–centroid distance is of 4.0755(7) Å and slippage of 1.384 Å. (**c**) Line-drawing for dinuclear fragment: M1 = M2 = Co (**1**); M1 = Co; M2 = Zn (**2**); (**d**) Schematic representation of 2D-CP, formed by interconnection of the ladder-like structures running along the *c*-axis; (**e**) *ab*-plane view of the structure showing the pores where other molecules can be housed (e.g., water molecules in the figure).

The results of the X-ray diffraction study for **1** and **2** are summarized in Table 1. The structure of {[CoCxAPy)]·2.15 H_2_O}_n_ (**1**) is depicted in Figure 1. It is closely related to previously reported complex {[ZnCxAPy)]·2CH_3_OH·0.125 H_2_O}_n_ [29]. These compounds crystallize in the same space group P-1 of triclinic system with very similar unit cell parameters and differ only by co-crystallized solvate molecules. Both complexes contain the same formula unit in the asymmetric part of the unit cell.

The asymmetric unit of **1** shows that each Co(II) atom is six-coordinated in a N_2_O_4_ strongly distorted octahedral geometry provided by four oxygen atoms from two μ_2_-к^2^O:O’ bridging and one к^2^O,O’ chelating carboxylate groups in equatorial plane and two nitrogen atoms from APy ligands in apical positions (Figure 1a). The octahedral distortion parameter calculated as the sum of the deviation from 90° for all 12 *cis* bond angles is of 74.82° (Table S2).

In a centrosymmetric dinuclear unit two Co(II) ions are bridged at Co1···Co1(1 − *x*, 1 − *y*, 1 − *z*) distance of 4.042(2) Å through two μ_2_-к^2^O:O’ carboxylate bridging ligands (Figure 1b,c). These dinuclear fragments are interconnected via four rigid 4,4’–azopyridine (APy) conforming a two-leg ladder structure running along the *c*-direction. The separation between Co ions within the ladder rails is 13.331(7) Å.

A 2D coordination framework within the *bc*-plane is formed by the connection of Co dimers of adjacent ladders through four flexible 1,3-bis(3-carboxypropyl)tetramethyldisiloxane linkers (Cx) in *trans*-oide conformation (Figure 1d). The Co···Co inter-ladder distance is 14.380(4) Å. The presence of offset face to face packing of adjacent APy ligands, which provide additional π-π stacking interactions, strengthens the 2D network. This interaction seems to be strong enough to determine the non-planarity of the fragment comprising two cobalt atoms, two chelating, and two bidentate-bridging carboxylate ligands (Figure 1d).

The crystal structure is constructed from the parallel packing of the discrete, weakly interacting 2D layers. The methyl groups located on both sides of the 2D plane act as a hydrophobic screen that prevents the development of strong 3D intermolecular interactions (Figure 1c). All the interlayer distances are equal or exceed the sum of the Van der Waals radii for the corresponding atoms. This packing displays infinite channels along the *c*-axis accommodating fractally disordered solvate molecules (Figure 1e). Further analysis of intermolecular interactions has revealed the lack of interlayer H-bonding involving co-crystallized water molecules. These structural features indicate that compound **1** is porous. The solvent accessible void fraction calculated, for empty channels, by the Olex2 routine (for probe radii of 1.2 Å and 0.2 Å) constitute 0.21 related to unit cell volume. 

According to X-ray crystallography, the heterometallic complex {[Zn_0.80_Co_0.20_CxAPy)]·1.5CH_3_OH}_n_ (**2**) (Table 1 and Table S3, Figures S7 and S8) is isostructural to **1**. Two metal ions in the dinuclear unit of **2** are joined via two μ_2_-к^2^O:O’ carboxylate bridging ligands at 3.9684(8) Å distances. The separation of metal ions across Cx and APy linkers is of 11.1000(9) Å and 13.3333(5) Å, respectively.

### 2.3. Theoretical Model and Ab Initio Calculations

The ground state of the free Co(II) ion is ^4^F (*L* = 3, *S* = 3/2). In the crystal, Co(II) ions are six-coordinated in a strongly distorted octahedral geometry, C_2v_ point group. Under the octahedral ligand field the single-ion ground state is the ^4^Γ_4_(^4^T_1_), which is split into three orbital singlets by the orthorhombic field terms, each with fourfold spin degeneracy. Finally, the spin-orbit coupling splits the multiplet into six Kramer’s doublets (KD’s) [34]. 

In most cases, only the ground doublet and the next excited doublet at an energy Δ are involved in the magnetic properties below room temperature. The spin-Hamiltonian formalism has been frequently used in the literature to describe the Zero Field Splitting (ZFS) of Co(II) complexes [35]. We have found this formalism adequate to describe magnetic properties of our complex below room temperature. The Hamiltonian describing single-ion properties in the presence of a magnetic field is given by:

(1) $$H_{SI}=D\left\{\left[S_{z}^{2}-\frac{1}{3}S\left(S+1\right)\right]+\eta \left[S_{x}^{2}-S_{y}^{2}\right]\right\}-\mu_{B}\vec{H}\hat{g}\vec{S}$$

The first two terms describe the orthorhombic Co(II) single ion anisotropy, where *D* and *η* = *E*/*D*, and the last term is the Zeeman term, with *g* the gyromagnetic factor for *S* = 3/2 spin. At low temperatures magnetic interactions may be of relevance to describing the magnetic properties, and an exchange interaction term needs to be added to the Hamiltonian. In the present case of a Co(II)-Co(II) dimer with Co(II) in high spin state, one deals with the case of ions showing unquenched orbital angular momentum. For this reason, the conventional Heisenberg-Dirac-Van Vleck (HDVV) model is not applicable. In an excellent review [36] it is shown that the exchange interaction is orbitally dependent, and the main effect of the non-quenched orbital moment on the dimer Hamiltonian may be expressed, whichever the different approximations are applied, in terms of a phenomenological or a microscopic pseudospin S* = 1/2 Hamiltonian, when the Hamiltonian is projected onto the subspace of low-lying Kramer’s doublets. Thus, we have proposed to use such a Hamiltonian in the present paper.

In {[CoCxAPy]·2.15H_2_O}_n_ the Co-Co dimer building block has a center of symmetry, which prohibits the antisymmetric interaction, on one hand, and Co^2+^ is highly anisotropic, on the other. Therefore, a dimer Hamiltonian with orthorhombic anisotropy, described by an axial anisotropy *D* and an orthorhombic component *E*, and Zeeman term has allowed us to describe magnetic properties of this compound for the range of temperatures up to 300 K:

(2) $$H_{dim}=\overset{2}{\underset{i=1}{\sum }}[DS_{i,z}^{2}+E\left(S_{i,x}^{2}-S_{i,y}^{2}\right)]-2J\left({\vec{S}}_{1}\cdot {\vec{S}}_{2}\right)-\mu_{B}\overset{2}{\underset{i=1}{\sum }}\vec{H}\hat{g}{\vec{S}}_{i}$$

operating on the dimeric *S* = 3/2⊗*S* = 3/2, 16-fold wave function base. The influence of orbital momentum is implicit in the principal values of local zero-field splitting tensor, on the one hand, and the local ***g***-tensors, on the other hand.

Following Palii’s receipt [36], at low temperature, the dimer anisotropic Hamiltonian acting on the S* = 1/2⊗S* = 1/2 four-fold dimer wave functions {½1/2, ±1/2 > |1/2, ±1/2 >} is:

(3) $$H_{dim}^{*}=-2\left(J_{x}^{*}S_{x}^{*1}S_{x}^{*2}+J_{y}^{*}S_{y}^{*1}S_{y}^{*2}+J_{z}^{*}S_{z}^{*1}S_{z}^{*2}\right)\;+{µ}_{B}\overset{2}{\underset{i=1}{\sum }}\left(g_{x}^{*}S_{x}^{*i}H_{x}+g_{y}^{*}S_{y}^{*i}H_{y}+g_{z}^{*}S_{z}^{*i}H_{z}\right)$$

where J*_α_ << *D*, α = x,y,z are the main axes of the g* and the J* tensors, which are coincident.

**Figure 2:**
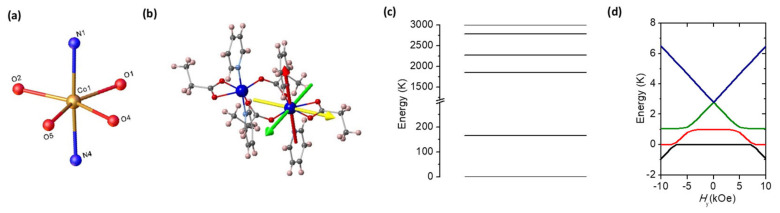
Single-ion properties. (**a**) Coordination sphere around Co(II) ion; (**b**) *ab initio* calculated easy axes of magnetization (the hard axis (HA, in red) is along the N-Co(II)-N axis; the easy axis (EA, in green) and intermediate axis (IA, in yellow) lie in the equatorial plane); (**c**) *ab initio* calculated energy levels of the six Kramer’s doublets; (**d**) energy level diagram for Co(II)-Co(II) S* = 1/2 dimer, with J*_x_/k_B_ = −0.5 K, J*_y_/k_B_ = −2.3 K and J*_z_/k_B_ = −0.5 K, as a function of magnetic field applied along easy axis.

In order to determine single-ion energy levels and identify an appropriate magnetic model, relativistic *ab initio* calculations have been performed, using the CASSCF/NEVPT2 method [37,38], as implemented in the ORCA 4.0 program [39,40]. In this method, the spin–orbit coupling and the spin–spin coupling relativistic effects, which are at the origin of the magnetic anisotropy, are included *a posteriori*. The *ab initio* calculations were done on a simplified version of the Co_2_ dimer fragment of compound **1** and **2**, where the Cx ligands were replaced by propionate and the APy ligands by pyridine (see Figure 2b) and where atomic positions were obtained from the experimental structure. In addition, one of the Co(II) ions was replaced by a diamagnetic Zn(II) ion. The basis-set was the DKH-Def2-TZVP [41] for all the atoms, which incorporates scalar relativistic effects. To speed up the calculations, the SARC/J auxiliary basis [41] along with the resolution of identity (RI) [42] and the chain-of-spheres (COSX) approximations [43] were used. In the CASSCF calculations, the active space was considered to be 10 Co(II) 3d and 3d’ orbitals containing 7 electrons (CASSCF(7,10)). Ten quartets and eleven doublets were used for the state-averaged CASSCF calculation. Then, the NEVPT2 calculations were performed with the CASSCF(7,10) reference space for the treatment of the dynamical correlation energy. After that, the effect of the spin–orbit coupling was taken into account using a mean-field operator (SOMF) [44,45], which was diagonalized on the basis of the previous CASSCF wavefunctions. After diagonalization of the *ab initio* g*-tensor for **1**, the eigenvalues were: *g*_1_* = 6.05, *g*_2_* = 3.82 and *g*_3_* = 1.91 (Table 2). The calculated Easy Axis of Magnetization (EAM) is depicted in Figure 2b. The hard axis is directed along the N-Co-N ligands axis, whereas the easy axis lies in the equatorial plane, near perpendicular to the Co-Co rung. The calculated ligand field is highly orthorhombic, with parameters η = *E*/*D* = 0.13, and *D*/k_B_ = 82 K. The calculated energy levels of the six KD’s are shown in Figure 2c. The energy gap between the ground KD and the first excited one is Δ/k_B_ = 166 K. Very similar parameters are obtained from *ab initio* calculations of the diluted complex, **2** (Table 2).

### 2.4. EPR Spectroscopy

**Figure 3:**
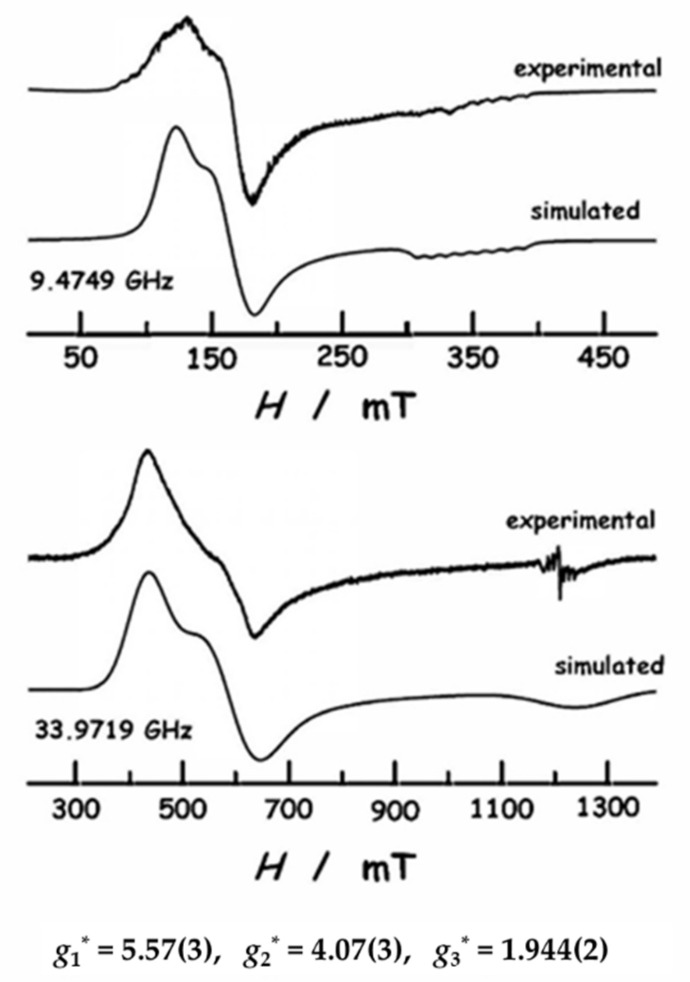
X-band (9.475 GHz, top) and Q-band (33.972 GHz, bottom) EPR spectra of the polycrystalline diluted sample **2** measured at *T* = 6 K, plotted as a function of magnetic field. Comparison of spectra with the simulation, calculated with EasySpin using the g* parameters given in the lower part of the graph and collected in Table 2.

EPR measurements on a polycrystalline sample of diluted compound **2** were performed to determine experimentally the anisotropic g* tensor principal values. Pure complex **1** is EPR silent, which may be indicative of a singlet ground state due to intra-dimer interactions; this will be shown below. Figure 3 displays the X-band and Q-band spectra measured at *T*= 6 K. Both spectra exhibit two features corresponding with the highest principal values of the g* tensor (*g*_1_* ≈ 5.6, *g*_2_* ≈ 4.1). On the other hand, the high field feature (*g*_3_* ≈ 1.9) is hardly observed in the Q-band spectrum, and overlaps with a signal that is due to the quartz tube sample-holder, separately measured. The simulated spectra, calculated with the help of EasySpin [46] using these principal values, are in good agreement with the experimental spectra.

As a summary, the dominant EPR signal can be described with an effective spin *S*_z_* = 1/2 with a gyromagnetic tensor with the principal values *g*_1_* = 5.57 ± 0.03, *g*_2_* = 4.07 ± 0.03 and *g*_3_* = 1.944 ± 0.002. The effective magnetic moment *µ* = 3.58 ± 0.08 µ_B_ was calculated as $\mu (\mu_{B})=g_{av}^{*}\sqrt{S^{*}(S^{*}+1)}$, with the average gyromagnetic moment, *g*_av_* = ((*g*_1_*^2^ + *g*_2_*^2^ + *g*_3_*^2^)/3)^1/2^ = 4.138. The predicted low temperature Curie factor is *C*_EPR_ = 1.61 ± 0.01 emu·K/mol.

As previously introduced, the single-ion ground state of Co(II) in a distorted octahedral ligand field preserves an important orbital contribution. Despite of this, we have made use of spin Hamiltonian formalism to describe magnetic properties up to room temperature. More elaborated models, which explicitly include spin orbit coupling, have been used in the literature to account for the effects of high energy levels [47]. However, such models have some limitations in the interdependency of the parameters and the unicity in fitting the magnetometry experimental results. Therefore, we deem appropriate the use of ZFS formalism in the present study.

Hence, the description of the two lowest Kramer’s doublets (KD1 and KD2) is given in terms of an effective spin *S* = 3/2 including a zero field term as described by Equation (1).

From the EPR point of view, given that the separation in energy of the two doublets Δ/k_B_ = 2*D*/k_B_(1 + 3η^2^)½ is much larger than the microwave energy, the system can be described by two S* = 1/2 systems with different effective g* tensors [48]. As it was obtained by *ab initio*, *D* > 0, therefore the ground KD1 corresponds to the *M*_S_ = ±1/2 components of the *S* = 3/2 and the principal values of the g* tensor are given by Pilbrow’s equations [49]:

(4) $$\begin{array}{ccc} g_{x}^{*}=g_{X}\left(1+\frac{1-3\eta }{\sqrt{1+3\eta^{2}}}\right) & & \\ g_{y}^{*}=g_{Y}\left(1+\frac{1+3\eta }{\sqrt{1+3\eta^{2}}}\right) & & \\ g_{z}^{*}=g_{Z}\left(\frac{2}{\sqrt{1+3\eta^{2}}}-1\right) & & \end{array}$$

which relate the apparent g*_x,y,z_ values within the ground state KD and the true *g*_x,y,z_ values of *S* = 3/2 as given in Equation (1) Hamiltonian. According to Equation (4), we may assign the EPR obtained gyromagnetic values, g*_1_, g*_2_, and g*_3_, to the corresponding g*_x_, g*_y_, and g*_z_ in the single-ion main axis system as g*_x_ = g*_2_ = 4.07 ± 0.03, g*_y_ = g*_1_ = 5.57 ± 0.03 and g*_z_ = g*_3_ = 1.944 ± 0.002.

Therefore, the positive *D* value is stabilizing in energy the lowest *M*_S_ doublet of the *S* = 3/2 multiplet. This implies that we have an easy plane anisotropy, consistent with *ab initio* calculations, where the axial *z*-axis is the hard magnetic axis, and the easy axis lies in the equatorial plane, along the *y*-axis.

### 2.5. Static Magnetic Properties (Up to Room T)

**Figure 4:**
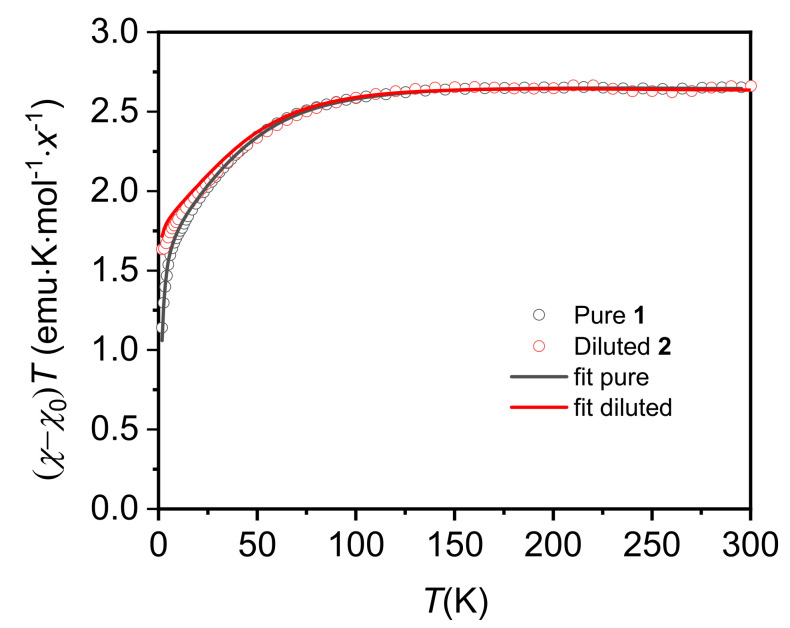
Temperature dependence of (*χ*−*χ*_0_)*T* for pure **1** (*x* = 1) and diluted compound **2** (*x* = 0.2), measured at *H* = 1 kOe. In line are shown the simulated curves as obtained from Equation (2) with the *D*, *E,* and *g*’s from the *ab initio* calculations of **1** and **2** and using *J* as a fitting parameter *J*/k_B_ = −0.32 K for **1**. Experimental data were corrected from a temperature independent term, $\chi_{0}$ = −7.0× 10^−4^ emu/mol for **1** and $\chi_{0}$ = −1.7× 10^−3^ for **2**.

Figure 4 plots the temperature dependence of *χ*·*T* for pure compound **1** and diluted complex **2**. Shown data have been corrected from a temperature independent susceptibility term, *χ*, (*χ*_0_= −7.0× 10^−4^ emu/mol for **1** and *χ*_0_ = −1.7× 10^−3^ emu/mol for **2**). This term accounts for the Van Vleck contribution of Co(II) high energy levels, and the dominant diamagnetic contribution of the Daphne Oil used to fix the sample grains (*χ_dia_* between −1× 10^−3^ and −2× 10^−3^ emu/mol). Both compounds show saturation to about 2.7 emu·K/mol at 300 K. This value is significantly higher than the theoretical spin-only value *χ*·*T* = 1.88 emu·K/mol predicted for an *S* = 3/2 (*g* = 2.00), indicating a relevant orbital contribution, as commonly observed for Co(II) compounds. As the temperature is lowered, *χ*·*T* continuously decreases due to the depopulation of electronic states. *χ*·*T* decreases faster below 100 K, reaching a value of 1.6 emu·K/mol at 2 K in the diluted compound, indicative of significant ZFS. This value is in perfect agreement with previously found low temperature Curie factor *C*_EPR_ = 1.61 ± 0.01 emu·K/mol. In the pure compound, *χ*·*T* tends to zero at very low temperatures, indicating the presence of antiferromagnetic interactions between the Co ions.

The temperature dependence of the magnetic susceptibility can be modeled with *ab initio* obtained energy levels and adjusted to parameters described by the spin Hamiltonian of Equation (2). Results are depicted in Figure 4 and compared with *χ*·*T* temperature dependence of pure **1** and diluted compound **2**.

Complementary, the magnetic susceptibility in the high temperature range, *T* > 150 K, has been analyzed in the frame of a Curie-Weiss law approximation

(5) $$\chi (T)=\chi_{0}+\frac{C}{\left(T-\theta \right)}$$

where *C* is the Curie constant, *χ*_0_ is the temperature independent contribution to the magnetic susceptibility and Curie temperature, *θ*, accounts for the magnetic interactions with the neighboring atoms. By fitting Equation (5) to the experimental data in the high temperature range (see Figure S9) the values shown in Table 3 were found. The obtained Curie constant, *C* = 2.67 emu·K/mol, corresponds to an effective magnetic moment *µ*_eff_ = 4.62 µ_B_, much larger than the spin-only contribution, *µ*_S_ = 3.87 µ_B_ (*S* = 3/2, *g* = 2.00), indicating an important orbital coupling. From this effective moment we deduce an average value for the gyromagnetic factor for the *S* = 3/2 four levels system, *g*_eff_ = 2.39.

Considering *g*_eff_^2^ = (*g*_x_^2^ + *g*_y_^2^ + *g*_z_^2^)/3, together with the relations given in Equation (4), yields a value for the orthorhombic factor of η = *E*/*D* = 0.165. Furthermore, we can determine the main values of the ***g***-tensor for the *S* = 3/2, *g*_x_ = 2.74, *g*_y_ = 2.28, and *g*_z_ = 2.11. These values largely deviate from the spin-only isotropic *g* = 2.00 confirming a significant spin-orbit contribution. Notably, the η = *E*/*D* factor is very similar to the value determined by *ab initio* calculations (see Table 2).

The Curie temperature *θ* = −1.1 K gives information of the sign and magnitude of the interaction in the dimer. Within the mean field theory, *θ* = 2z*JS*(*S* + 1)/3k_B_, where *z* is the nearest neighbors number and *J* is the average interaction. For a *S* = 3/2 dimer, *z* = 1, with exchange interaction H = −2*J*S.S, we obtain an average AF interaction in the Co(II) dimer of *J*/k_B_ = −0.44 K, close to the exchange interaction used to fit *χ*·*T* curve of 1 with *ab initio* parameters, *J*/k_B_ = −0.32 K.

### 2.6. Static Magnetic Properties (T < 20 K)

**Figure 5:**
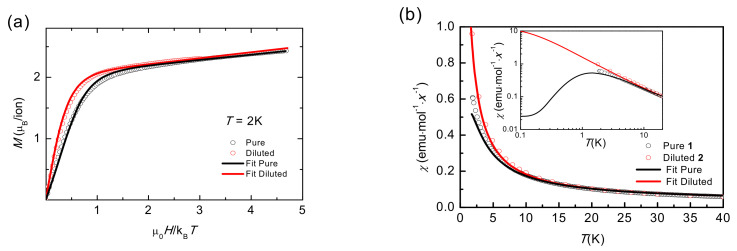
(**a**) Field dependence of the magnetization, *M*(*H*), measured at *T* = 2 K for **1** and **2**. (**b**) Low temperature detail of magnetic susceptibility *χ*(*T*) data at 1. kOe for **1** (*x* = 1) and **2** (*x* = 0.2). The lines correspond to fits with Equation (3) using the g* components deduced from the EPR measurements, the intra-rung anisotropic exchange constants J*_x_/k_B_ = −0.5 K, J*_y_/k_B_ = −2.3 K, and J*_z_/k_B_ = −0.5 K, and integrating over the randomly oriented grains. A Van Vleck contribution is added to the magnetization simulated curves. For the diluted compound the dimer exchange interaction is not considered in the calculation.

The field dependence of the magnetization of a powdered sample measured at *T* = 2.0 K up to 140 kOe is shown in Figure 5a for pure **1** and diluted **2** compounds. The magnetization per Co ion is represented as a function of reduced µ_0_*H*/k_B_*T*. The *M*(*H*) dependence for a powder, spatially averaged moment, has been calculated, according with Equation (3), with the g* tensor parameters obtained by EPR (g*_x_ = 4.07, g*_y_ = 5.57, g*_z_ = 1.944). A Van Vleck contribution, *χ*_TI_ = 1.8(1)·10^−2^ emu·mol^−1^, was added to fit the experimental *M*(*H*) data. The simulated curve fits perfectly the experimental data for the diluted compound **2**, where the exchange interaction is negligible. The agreement is satisfactory and proves the adequacy of using the S* = 1/2 approximation at low temperatures, on one hand, and that the Co(II) ground state has an orthorhombic anisotropy, on the other hand. The *M*(*H*) curve for pure **1** lies below that of the diluted compound **2**, which can be attributed to an AF interaction within the Co(II) dimer. Indeed, a good fit of the *M*(*H*) for the pure compound was achieved with the above g* single-ion tensor components, and the dimeric anisotropic exchange components J*_x_/k_B_ = −0.5 K, J*_y_/k_B_ = −2.3 K, and J*_z_/k_B_ = −0.5 K, which are the best-fit parameters of low temperature *χ*(*T*), *M*(*H*), and heat capacity analysis (*vide infra*).

The adequacy of the dimer model and its anisotropic character is further corroborated by the simulation of the low temperature magnetic susceptibility *χ*(*T*). Figure 4 shows the experimental data for pure **1** and diluted **2** compounds together with simulations. The dimer model perfectly fits the susceptibility for **1**. For AF coupling, the *χ*(*T*) shows a maximum at a temperature of the order of the interaction, as shown in Figure 5b inset, not observed experimentally since it falls below the measured temperature range. Furthermore, when the interaction is canceled, as for the diluted compound **2** the curve is perfectly simulated with the Zeeman term as indicated in Equation (3).

### 2.7. Heat Capacity

**Figure 6:**
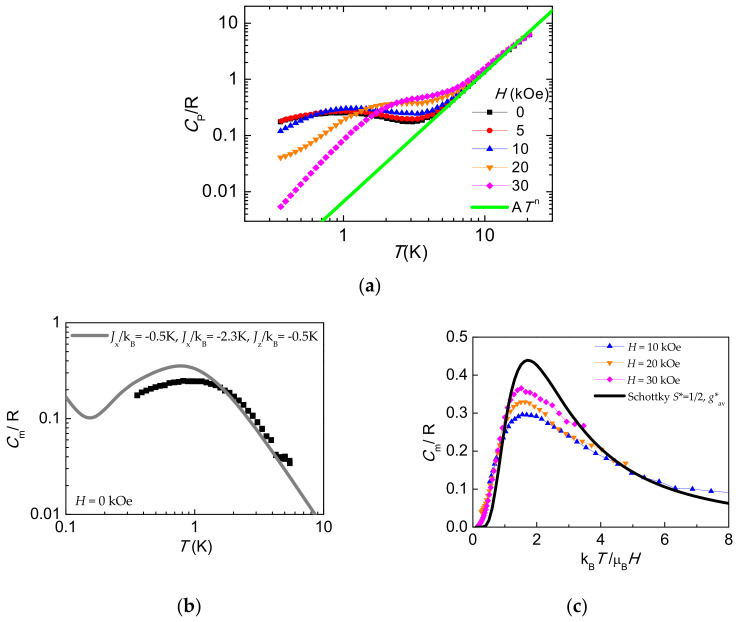
(**a**) Heat capacity as a function of temperature for various applied fields for compound **1**. The lattice contribution is depicted by the solid green line. (**b**) Magnetic contribution to the heat capacity as a function of temperature for *H* = 0. The solid grey line is the simulated *C*_m_(*T*) curve as obtained within the dimer model given in Equation (3), with anisotropic AF exchange constants, J*_x_/k_B_ = −0.5 ± 0.05 K, J*_y_/k_B_ = −2.3 ± 0.3 K, and J*_z_/k_B_ = −0.5 ± 0.05 K plus a hyperfine contribution. (**c**) Magnetic heat capacity contribution as a function of the adimensional *x* = k_B_*T*/μ_B_*H*. Continuous black line: calculated two-level Schottky curve with g*_av_ = 4.138.

The measurement of heat capacity is not commonly used in the study of magnetic molecules although it can be complementary and provide valuable information on electronic levels and the energy of phonons, as well as magnetic interactions [50]. In some cases the determination of the low temperature heat capacity has been key to determining the slow relaxation mechanisms in SMM metal complexes [51]. In the present case, seeking to obtain more information of the magnetic interactions, the heat capacity (HC) of a powdered sample of compound **1** was measured as a function of the temperature, under different applied fields ranging from 0 < *H* < 30 kOe (Figure 6a). The lattice contribution, *C_L_*/*R* = *αT*^−*n*^, α = 6.35 × 10^−3^ K^n^, *n* = 2.3, dominated at high temperatures (*T* > 10 K), and was subtracted from the experimental data to obtain the magnetic contribution, *C*_m_/R, which is shown in Figure 6b,c.

The magnetic heat capacity at zero field displays a rounded contribution centered around 1 K which may be attributed to low dimensional or short range magnetic ordering. Although the curve is featureless, the broadness and extension indicates that it has to comprise different contributions, which cannot be accounted for with a simple model. In view of the structure of the compound, where the separation between the intra-rung Co-Co ions is much smaller than the inter-rung separation, a magnetic dimer model with anisotropic exchange interaction, as defined by Equation (3), was adopted to fit the magnetic contribution to the heat capacity. The best compromise in reproducing the *C*_m_(*T*) at *H* = 0 kOe curve (see Figure 6b), together with *M*(*H*) at 2 K and *χ*(*T*) at low *T*, was obtained for the following set of exchange parameters, J*_x_/k_B_ = −0.5 ± 0.05 K, J*_y_/k_B_ = −2.3 ± 0.3 K, and J*_z_/k_B_ = −0.5 ± 0.05 K, which are fully compatible with the orthorhombic character of the Co(II) ground state. Indeed, the anisotropic exchange interaction splits the four energy levels of the dimer (*S*_1_⊗ *S*_2_ = 0, 1) (see Figure 2d), giving as a result a broad contribution of the HC at zero magnetic field. The change in magnetic entropy below 10 K, reaching 0.5–0.6 R per Co ion supports this model (see Figure S10). 

When a magnetic field is applied, the exchanged split levels of Co(II) dimer are further separated, and at high magnetic field the *C*_m_(*T*,*H*) curves are dominated by the Zeeman term. They converge to a two-level Schottky anomaly with EPR g*_av_ = ((g*_x_^2^ + g*_y_^2^ + g*_z_^2^)/3)^1/2^ = 4.138 as illustrated in Figure 6c where the *C*_m_(*T*) at different fields are plotted as a function of the adimensional parameter k_B_*T*/µ_B_*H*.

At very low temperatures the hyperfine contribution (hf) to the heat capacity has to be taken into account. The natural abundance of the *I* = 7/2 isotope (^59^Co) is 100% and its nuclear moment is *µ* = 4.613 µ_N_. The hyperfine heat capacity at high temperatures may be approximated to:

(6) $$C_{hf}/R=\frac{A^{2}I(I+1)}{12k_{B}^{2}}\frac{1}{T^{2}}=\frac{b}{T^{2}}$$

where *A* is the average hf interaction constant formulated for a S* = ½. A value of *b* = 1.66× 10^−3^ K^2^ [52] has been considered in the simulation, similar to other values reported in the literature for Co organic complexes [53]. The hyperfine contribution is much smaller than the spin exchange interaction, introducing additional degrees of freedom and splitting the electronic levels at very low temperature.

### 2.8. Dynamic Magnetic Properties

Magnetic relaxation phenomena of compound **1** were explored by ac susceptibility measurements as a function of frequency, dc magnetic field strength, and temperature.

**Figure 7:**
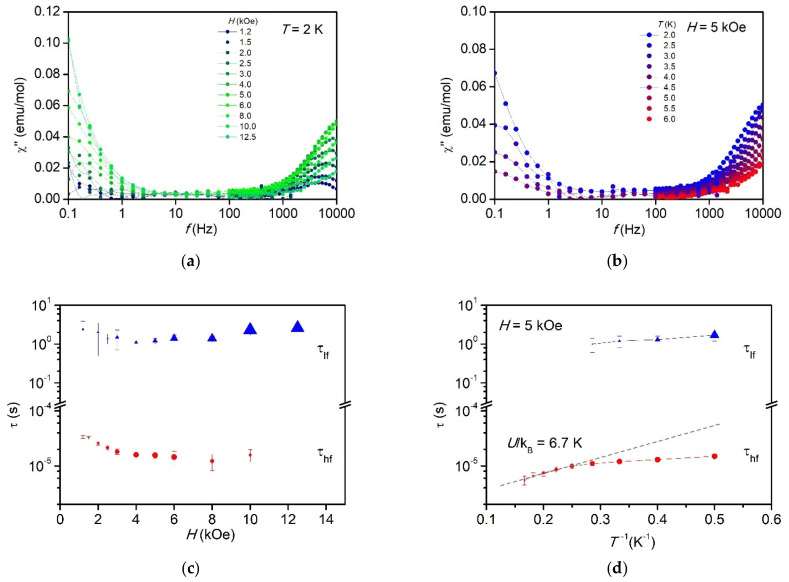
Magnetic relaxation for compound **1**. Top: Out-of-phase component of the susceptibility as function of frequency, (**a**) at constant *T* = 2 K and diverse applied fields, and (**b**) at constant field *H* = 5 kOe and different temperatures. Bottom: spin relaxation time as a function of the field, τ(*H*) at *T* = 2 K (**c**), and as a function of the inverse temperature, τ(1/*T*) at *H* = 5 kOe (**d**), for the two different observed processes, τ_lf_ and τ_hf_.

No out-of-phase signal (*χ*″ = 0) was observed in the measured frequency range, from 0.1 Hz to 10 kHz, at zero dc magnetic field indicating an isothermal response of the spin system through fast spin relaxation. However, when an external dc field was applied an increase of the *χ*″ signal was observed for fields larger than *H* = 1 kOe (see Figure 7a,b). Two different field-induced slow relaxation of the magnetization processes were well distinguished, one at very low frequencies at the edge of the lower-frequency measurement window, and another process lying at the high-frequency edge. Given that it is a randomly oriented sample, it may be inferred that the anisotropic spin system participates in one or another mechanism depending on the relative orientation with respect to the external magnetic field. Other faster relaxation pathways are not excluded.

Since for most of the *χ*″(*f*) curves no maximum was attained within experimental frequency range, the estimation of the relaxation times was obtained from the expression [50]:

(7) $$\tau (T,H)=\frac{\chi^{\prime \prime }(f,T,H)}{\left(\chi^{\prime }(f,T,H)-\chi_{\infty }(T,H)\right)\cdot 2\pi f}$$

where *χ*_∞_ is the adiabatic susceptibility at the limit of high frequency (*f* → ∞), when spins are not responding to magnetic field. For the low frequency process (*f* < 1 Hz), the adiabatic susceptibility can be easily determined by the value at 100 Hz, *χ*_∞_^lf^ = *χ*′(100 Hz). Note that 100 Hz can be considered as high frequency in comparison to *f* < 1 Hz. For the high frequency process the determination of the adiabatic susceptibility was more intricate (see SI.4 for details).

The obtained relaxation times for the two processes, τ_lf_ and τ_hf_, as function of the magnetic field and the inverse of the temperature are depicted in Figure 7c,d, respectively. The low frequency relaxation time is nearly independent on the magnetic field and temperature, τ_lf_ ≈ 2 s. It is a likely process only at the lowest temperatures and increasing in amplitude with field, i.e., the number of centers exhibiting slow relaxation and being blocked at high frequencies increases with increasing magnetic field. On the other hand, the high frequency relaxation process has a slight dependence with field, decreasing the relaxation time as field increases.

The fit of the high temperature range of τ_hf_(1/*T*) to an Arrhenius law, τ = *τ*_0._exp(*U*/k_B_*T*), affords an effective energy barrier for the reversal of the magnetization of *U*/k_B_ = 6.7 ± 0.5K and prefactor *τ_0_* = 1.9 ± 0.2× 10^−6^ s. This energy is of the order of the energy separation between states within the magnetic dimer model (see Figure 2d). Thus, this process is ascribed to an Orbach mechanism within the dimer levels. At low temperatures, the relaxation time evolves to a direct process mechanism. Attempts to reproduce the temperature dependence of the high frequency process assuming a direct process and a Raman mechanism were unsuccessful.

The observation of a very slow process at low temperatures indicates a partial blockage of the magnetization in coexistence with a faster relaxation mechanism. The obtained relaxation times, although of the order of seconds, are not slow enough to observe the opening of the hysteresis loop at 1.8 K at the fastest speed achievable in our experimental system, 180 Oe/s.

## 3. Discussion

In compound **1**, the interaction path in the dimer rung is via two carboxylate bridges Co-O-C-O-Co. Most, if not all, of the reported exchange interactions in carboxylate bridged Co-Co complexes are antiferromagnetic [15]. However, its strength depends on the modes of connection (syn–syn, syn–anti, or anti–anti), the angles, and also on the Co-Co distance.

To check this in more detail, in Table 4 Co-Co dimers with carboxylate exchange paths bridging both metal atoms have been collected. Furthermore, those compounds with carboxylate and oxygen mediated bridges are included. As a third group, two cases with oxalate bridges have been also included for comparison’s sake.

The local coordination in **1** is a distorted octahedron CoN_2_O_4_, with four oxygen atoms in the equatorial plane and two apical N atoms, which gives rise to a positive anisotropy constant *D*/k_B_ = 82 K with an orthorhombic component *E*/*D* = 0.13. The most similar cases, from the viewpoint of the coordination and bridging paths are [Co(Htatb)(bimbp)].DMF, and [Co(Htatb)(1-3-bimbp)].DMF [54]. The three compounds have just 2 Co-O-C-O-Co syn-syn bridges, giving rise to an exchange interaction constant ranging between *J*/k_B_ = −6.65 and −2.3 K, that is, a weak antiferromagnetic interaction. No information on their single-ion anisotropy is available on the latter two cases. The other compounds with two or one carboxylate bridges also depict a different Co coordination, either with two N atoms, one adjacent to the other, or all oxygen atoms. The exchange interaction decays strongly in those compounds in comparison to the present compound.

It is of some interest to mention the compound Co_2_(esp)_2_(EtOH)_2_, with a very different coordination, namely fourfold pyramidal, with the ligand at the apex. In contrast to all other cases, its anisotropy constant *D* < 0, i.e., axial easy axis. The Co-Co distance is very short, probably indicating the presence of a contribution from direct metal-metal interaction to the exchange interaction. Additionally, it supports four carboxylate bridges. Both contributions generate an AF exchange of *J*/k_B_ = −9.49 K [55].

In stark contrast, the compounds with one or two oxygen mediated paths have ferro- or antiferromagnetic exchange, as high as *J*/k_B_ = 26 K in [Co_2_(μ-OAc)_3_(urea)(tmen)_2_][OTf] [56]. Thus, the oxygen mediated bridge may modify substantially the Co-Co interaction.

We have also included a last class of selected dimers, with oxalate bridging ligand, for comparison purposes. As said above, in the present work the magnetic properties of {[CoCxAPy]⋅2.15H_2_O}_n_ have been interpreted in terms of a two-legged ladder with predominant antiferromagnetic intra-rung interaction and with negligible intra-leg interaction. A similar conclusion was found in the compound Na_2_Co_2_(C_2_O_4_)_3_(H_2_O)_2_, where anisotropic Co^2+^ ions form a *S* = 1/2 ladder, albeit with antiferromagnetic rung interaction; after neutron inelastic scattering experiments proved the predominance of the dimeric character of the dispersion relation [57]. At low temperature the anisotropic exchange Hamiltonian is applied, with a relatively strong intra-rung interaction *J*/k_B_ = −30.5 K, and an exchange anisotropy of *J*_x,y_/*J* ≅ 0.35 K. This case is of interest since the authors have applied the same approach as we have done in {[CoCxAPy]·2.15H_2_O}_n_.

In Table 5 we have shortlisted the reported Co(II) dimers which entail SMM behavior. To be noticed is that there is no example of a Co(II) dimer showing slow relaxation at *H* = 0. Only some Co(II) dimers show slow magnetic relaxation under a field, and remarkably, all previously reported, have intradimer ferromagnetic exchange. Thus, to our knowledge, the present compound is the first Co(II) antiferromagnetically coupled dimer showing slow relaxation of the magnetization.

At zero magnetic field we do not observe spin relaxation of the magnetization within our frequency experimental window. This result may indicate that spin relaxation is very fast, and we are observing the isothermal susceptibility, what could be explained as due to a strong spin coupling through hyperfine interactions. The nuclear moment *I* = 7/2 of ^59^Co couples with the S* = 1/2 ground state dimer levels leading to the opening of new relaxation paths. A sizable Van Vleck susceptibility ensues giving rise to nearly frequency independent *χ*’ susceptibility. As the magnetic field increases there is a continuous crossover giving the states a net magnetic moment that makes them detectable by magnetic susceptibility measurements. Therefore, slow relaxation can be observed under a magnetic field of sufficient intensity. This mechanism is described for a single Co(II) ion [79]. However, it necessarily is also taking place in the dimer; in our antiferromagnetically coupled dimer model, we are dealing with a singlet ground state with zero expected magnetic moment for *H* = 0. A small ac magnetic field would induce a reversible build-up of magnetic moment by modulating the electronuclear wave function, in a so called Van Vleck susceptibility component. This component does not drive the spin system out of equilibrium and therefore does not contribute to spin relaxation processes.

As a magnetic field is applied, the dominant AF interaction and the anisotropy in the interaction gives as the result four levels with a weak dependence with the applied field (see Figure 2d). This would explain the need for a rather large field to show a sizeable change, and the appearance of slow relaxation processes for fields above 1.2 kOe.

On the other hand, in order to gain insight into the spin relaxation phenomena we analyzed the relative weight of the different observed processes. If we take a closer look at the ac susceptibility variation with the magnetic field at *T* = 2 K, it is remarkable the strong variation of the adiabatic susceptibility of the slow frequency process, which is determined by the *χ*’ at 100 Hz, *χ*_∞_^lf^ (see Supplementary Materials, Figures S11 and S12). This is explained by the fact that the larger the field, the more dominant is this process with respect to the reversible susceptibility contribution, as more effective moment is grown in the ground state. For *H* = 5 kOe, 22% of the magnetic susceptibility is blocked for frequencies larger than 1 Hz, which increases to a 52% for 10 kOe. We have assigned this long relaxation time process to a direct process.

It is worthy to note that in our complex the dimers are assembled in 2 leg infinite ladders (2LL) along the *c*-axis. This configuration makes this compound a potential candidate as a physical realization of a 1D Haldane gap system, for which interesting theoretical predictions have been made [80].

We have demonstrated that in the investigated temperature range (down to 1.9 K) the static and dynamic magnetic data may be rationalized in terms of a dimeric model of Co-Co with intra-rung interaction, with negligible leg-interaction.

Nevertheless, it is plausible that at lower temperatures intra-leg interactions would start to be relevant, and manifest in the way the magnetic susceptibility decays as temperature decreases towards *T* = 0. Indeed, at low temperatures, the magnetic sublattice can be modelled as a 2LL *S* = 1/2 symmetric ladder, with the Co-Co rung-interaction, *J*_⊥_, and the intra-leg interaction, *J*_II_. The magnitude and sign of the ratio *J*_⊥_/*J*_II_ determine a large variety of collective magnetic behavior (*J*_⊥_ < 0, [81] *J*_⊥_ > 0 [82]). Of importance is whether the ladder is a Haldane gap system, since in that case the magnetic susceptibility approaches zero at *T* = 0 in the Haldane gapped model, while the magnetic susceptibility decreases to finite values in the case of no-gap.

In terms of the ladder model, the present case corresponds to approximately *J*_⊥_/ *J*_//_ →+∞, where *J*_⊥_ has AF character. In Shelton et al. 1996 [83], it was shown that a *S* = 1/2, 2LL spin-ladder always has a spin-gap irrespective of the sign of *J*_⊥_, and proportional to it. In the *J*_⊥_ < 0 (AF case), the ground state is a singlet, and the ladder excited band is the dimer *S* = 1 triplet with a width ≈ *J*_//_. In the *J*_⊥_ > 0 (FM case), the ground state is a triplet, and the system behaves as a *S* = 1 linear chain, and, therefore, has a Haldane spin-gap.

Therefore, in view of the anisotropic interaction and the topology, this complex may result in a particular and interesting realization of a Haldane gap. For the study of this model, and their interesting theoretical predictions, we would need to go down to much lower temperatures (*T* < 0.4 K), where AF interdimer interaction along the 2LL may start to play a role as the kinetic energy becomes of the order of the leg weak interaction.

## 4. Materials and Methods

### 4.1. Experimental Techniques

Fourier transform infrared (FTIR) spectra were recorded using a Bruker Vertex 70 FTIR spectrometer. Registrations were performed in the transmission mode in the range 400−4000 cm^−1^ at room temperature with a resolution of 2 cm^−1^ and accumulation of 32 scans. UV-Vis absorption spectra measurements were carried out in DMF solution on a Specord 200 spectrophotometer. Carbon, hydrogen, and nitrogen content were determined on a Perkin–Elmer CHNS 2400 II elemental analyzer. Energy-dispersive X-ray spectroscopy (EDX) system available on the Quanta 200 scanning electron microscope (FEI company, Brno, Czech Republic) and X-ray fluorescence (EDXRF) system EX-2600 X-Calibur SDD (Xenemetrix, Migdal HaEmek, Israel) were used to estimate the Zn:Co molar ratio in compound **2**. The thermogravimetric analyses were performed on a STA 449F1 Jupiter NETZSCH (Germany) equipment within 20–700 °C under a nitrogen flow (50 mL/min) using a heating rate of 10 ^o^C/min, taking 10 mg sample. Alumina crucible was used as sample holder. Differential scanning calorimetry (DSC) measurements were conducted on a DSC 200 F3 Maia (Netzsch, Germany) at a heating/cooling rate of 10 °C/min in nitrogen used as inert atmosphere at a flow rate of 100 mL/min.

X-ray diffraction data for **1** and **2** were collected with an Oxford-Diffraction XCALIBUR E CCD diffractometer equipped with graphite-monochromated MoKα radiation. The experimental data comprise 383 and 388 frames each for 5 and 180 s over 1° oscillation width and crystal-to-detector distance at 40 mm. The unit cell determination and data integration were carried out using the CrysAlis package of Oxford Diffraction [84]. The structure was solved by direct methods using Olex2.77 software with the SHELXS structure solution program and refined by full-matrix least-squares on F² with SHELXL-2015 [85]. Hydrogen atoms were inserted in fixed, idealized positions and refined as rigidly bonded to the corresponding non-hydrogen atoms. The positional parameters for H-atoms of OH groups were determined from Fourier maps and refined according to H-bonds parameters. Identical positional parameters and displacement parameters were assumed for Co and Zn atoms in the crystal 2. When the occupancy factors for Co and Zn atoms were allowed to be refined, a conclusive ratio of 0.82:0.18 has resulted. The molecular plots were obtained using the Olex2 program. Crystal data and some further details concerning X-ray analysis are given in Table 1. The bond lengths and angles for 1 and 2 are listed in Tables S2 and S3, CCDC- 2005545, 2005606. These data can be obtained free of charge via www.ccdc.cam.ac.uk/conts/retrieving.html (or from the Cambridge Crystallographic Data Centre, 12 Union Road, Cambridge CB2 1EZ, UK; Fax: (+44) 1223-336-033; or structure@ccdc.ca.ac.uk). Powder X-ray diffractogram was recorded on a Rigaku Miniflex 600 diffractometer (Tokyo, Japan) using CuK_α_-emission in the angular range of 10–60 (2*θ*) with a scanning step of 0.01^o^ and a recording rate of 1°/min.

Electron Paramagnetic Resonance (EPR) measurements were conducted in a ELEXYS E580E spectrometer from Bruker. A liquid helium refrigerated Oxford CF900 continuous-flow cryostat was employed for measurements at low temperatures. The powdered polycrystalline sample was dispersed in n-hexane and introduced in a quartz tube (707-SQ from Wilmad Labglass). The sample was cooled at zero field to avoid preferential orientation in the field.

Static magnetic measurements, magnetization, *M*(*H*), and susceptibility, *χ*(*T*), data were collected from polycrystalline samples, embedded in Daphne Oil or n-Hexane to avoid grain orientation, in a Quantum Design SQUID Magnetometer equipped with the RSO option and a Quantum Design PPMS with the VSM option. *M*(*H*) was measured at *T* = 2.0 K up to 140 kOe and *χ*(*T*) from 1.9 to 300 K, with an applied field of 1 kOe. Heat capacity under different applied fields (0–30 kOe) was measured down to 0.35 K on a pressed powder pellet fixed with Apiezon N grease, using the same PPMS equipped with a ^3^He refrigerator.

Dynamic magnetic measurements for polycrystalline samples were done at fixed temperatures in the range 1.8 < *T* < 6 K, with an excitation field of 4 Oe, at dc bias fields in the range 0 < *H* < 50 kOe, while sweeping the frequency 0.1 < *f* < 10,000 Hz. Low frequency range (0.1 Hz–1 KHz) was measured with a SQUID magnetometer, and high frequency range (10 Hz–10 kHz) with the ACMS magnetometer option of the PPMS.

### 4.2. Materials

1,3-Bis(3-carboxypropyl)tetramethyldisiloxane (H_2_Cx), [HOOC(CH_2_)_3_(CH_3_)_2_Si]_2_O, MW = 306.5 was prepared according to already published procedure [86], while 4,4′-azopyridine (APy), C_10_H_8_N_4_, MW = 184.20, Cobalt(II) perchlorate hexahydrate, Co(ClO_4_)_2_·6H_2_O, MW = 365.93 and Zinc perchlorate hexahydrate, Zn(ClO_4_)_2_·6H_2_O, MW = 372.38, 2,6-Dimethylpyridine, C_7_H_9_N, MW = 107.15, and ethanol were purchased from Sigma-Aldrich.

#### 4.2.1. Preparation of {[CoCxAPy)]·2.15 H_2_O}_n_ (**1**)

1,3-Bis(3-carboxypropyl)tetramethyldisiloxane, Cx, (0.150 g, 0.49 mmol) was mixed with 4,4′-azopyridine, APy, (0.090 g, 0.49 mmol) in 10 mL ethanol. The mixture was stirred 20 min and, after adding 2,6-dimethylpyridine (0.053 g, 0.49 mmol), was heated to reflux for one hour. After cooling to room temperature, this mixture was filtered and added by layering on the walls of the glass tube containing a solution of 0.360 g (0.98 mmol) Co(ClO_4_)_2_x6H_2_O dissolved in 15 mL distilled water. It was left to rest at room temperature. In these conditions, a crystalline compound **1** is obtained; Yield: 0.178 g, 31% based on Co content, separated from the reaction mixture after more than two weeks. Elem. anal., wt%: Calcd. for C_22_H_36.3_CoN_4_O_7.15_Si_2_ (MW = 586.36): C 45.06, H 6.24, N 9.56. Found: C 44.93, H 6.50, N 9.98. IR ν_max_ (KBr), cm^−1^: 3545 s, 3418 s, 3105 w, 2953 m, 2922 m, 2895 m, 2872 w, 1724 w, 1601 s, 1555 vs, 1483 w, 1447 s, 1425 s, 1385 s, 1319 m, 1254 s, 1225 m, 1124 w, 1083 s, 1047 vs, 1016 s, 876 w, 839 s, 795 s, 748 w, 700 w, 665 w, 623 s, 585 w, 571 m, 549 w, 527 w.

#### 4.2.2. Preparation of {[Zn_0.80_Co_0.2_CxAPy)]·1.5 CH_3_OH}_n_ (**2**)

Compound **2**, wherein Co was largely replaced by zinc was prepared under similar conditions but in methanol as a solvent (10 mL) and using a mixture Co(ClO_4_)_2_·6H_2_O: Zn(ClO_4_)_2_·6H_2_O at molar ratio 5:95, namely 0.018 g (0.05 mmol) Co(ClO_4_)_2_·6H_2_O and 0.354 g (0.95 mmol) Zn(ClO_4_)_2_·6H_2_O, the quantities of the other reactants and procedure remaining the same as in the recipe of compound 1; Yield: 0.026 g, 17% based on Co content. Elem. anal., wt%: Calcd. for C_23.5_H_38_Co_0.2_N_4_O_6.5_Si_2_Zn_0.8_ (MW = 600.81): C 46.98, H 6.38, N 9.33. Found: C 45.50, H 6.24, N 9.78. Co:Zn molar ratio: 0.22:0.78 (EDX), 0.21:0.79 (XRF); IR ν_max_ (KBr), cm^−1^: 3437 m, 3105 vw, 3076 vw, 3044 vw, 2953 m, 2922 m, 2893 m, 2870 w, 2795 vw, 1713 w, 1601 vs, 1553 vs, 1481 w, 1445 s, 1423 s, 1339 w, 1317 m,1254 s, 1223 m, 1165 w, 1126 m, 1045 vs, 1016 s, 968 w, 880 w, 837 s, 795 s, 744 m,700 m, 663 w, 606 w, 571 m, 548 m, 528 m.

## 5. Conclusions

The synthesis, structure, and magnetic properties of a new MOF compound, {[CoCxApy]·2.15 H_2_O}_n_, built from the stacking of 2D CPs formed by the adjacent bonding of ladders with dimer Co-rungs have been reported. A multitechnique approach, based on EPR, heat capacity, and magnetic characterization, together with *ab initio* calculations, has allowed the unequivocal determination of the magnetic model and the exchange coupling scheme. Single-ion Co(II) in this compound is described by two KD’s separated 166 K in energy, where the *M*_S_ = ±1/2 doublet is stabilized as ground state with effective gyromagnetic factors *g*_x_* = 4.07, *g*_y_* = 5.57, and *g*_z_* = 1.944. Co(II) ions form magnetic dimers with anisotropic AF exchange parameters, J*_x_/k_B_ = −0.5 ± 0.05 K, J*_y_/ k_B_ = −2.3 ± 0.3 K, and J*_z_/ k_B_ = −0.5 ± 0.05 K. The degree of anisotropy in the magnetic exchange is of similar magnitude as the anisotropy of the Co(II) single-ion ground state. The Co(II) dimer shows SMM behavior under applied magnetic field. Two different magnetic relaxation processes show up, a slow _lf_ ≈ 2 s direct process and an Orbach process at higher frequencies with *U*/k_B_ = 6.7 ± 0.5 K. Slow relaxation processes involve uniquely dimer energy levels. The new compound represents a rare instance of a MOF built up from ladders showing SMM of the dimeric Co-rungs.

## Figures and Tables

**Table 1:** Crystallographic Data.

	1	2
CCDC	2005545	2005606
Empirical formula	C_22_H_36.3_CoN_4_O_7.15_Si_2_	C_23.5_H_38_Co_0.2_N_4_O_6.5_Si_2_Zn_0.80_
Formula weight	586.36	600.81
Temperature/K	200	200
Crystal system	triclinic	triclinic
Space group	*P*-1	*P*-1
*a*/Å	11.8234(13)	11.7865(9)
*b*/Å	11.8324(13)	11.8192(4)
*c*/Å	13.3262(11)	13.3333(5)
*α/°*	110.597(8)	110.367(4)
*β*/°	96.107(8)	96.376(5)
*γ/°*	114.090(10)	114.261(5)
*V*/Å^3^	1523.5	1515.77(15)
*Z*	2	2
*D*_calc_/mg/mm^3^	1.278	1.316
*μ*/mm^−1^	0.685	0.882
Crystal size/mm^3^	0.15 × 0.10 × 0.10	0.30 × 0.10 × 0.05
*θ*_min,_ *θ*_max_(^°^)	4.938 to 50.048	3.97 to 50.052
Reflections collected	10714	11780
Independent reflections	5309 [*R*_int_ = 0.0576]	5329 [*R*_int_ = 0.0300]
Data/restraints/parameters	5309/0/339	5329/0/350
*R*_1_*^a^*(*I* > 2σ(*I*)	0.0829	0.0438
*wR*_2_*^b^*(all data)	0.2221	0.1198
GOF *^c^*	1.022	1.058
Largest diff. peak/hole/e Å^−3^	1.21/−0.56	1.01/−0.30

^a^*R*_1_ = Σ‖*F*_o_|−|*F*_c_‖/Σ|*F*_o_|. ^b^ *wR*_2_ = {Σ[*w*(*F*_o_^2^−*F*_c_^2^)^2^]/Σ[*w*(*F*_o_^2^)^2^]}^1/2^. ^c^ GOF = {Σ[*w*(*F*_o_^2^−*F*_c_^2^)^2^]/(*n*−*p*)}^1/2^, where *F*_a_ and *F*_c_ are the observed and calculated structural factors, respectively, *n* is the number of reflections and *p* is the total number of parameters refined.

**Table 2:** Summary of parameters of single-ion ZFS Hamiltonian given by Equation (1). *ab initio* and experimental, together with S* = ½ ground state KD1 g*-factors.

		*Ab initio/Fit* *Compound 1*	*Ab initio* *Compound 2*	*Experimental*
*S* = 3/2	*D*/k_B_(K)	82.0	83.0	
	η = *E*/*D*	0.13	0.20	0.165(5)
	*J*/k_B_(K)	−0.32		−0.44
	*g*_x_ *g*_y_ *g*_z_	2.40 2.58 1.96	2.37 2.64 1.95	2.74(3) 2.28(2) 2.11(1)
S* = 1/2	*g*_x_* g_y_* g_z_*	3.82 6.05 1.91	3.28 6.58 1.79	4.07(3) 5.57(3) 1.944(2)

**Table 3:** Fit parameters of *χ*−^1^*(T)* data to a Curie-Weiss law.

	Pure 1	Diluted 2
*χ*_0_ (emu·mol^−1^)	−7.0·10^−4^	−1.7·10^−3^
*C* (emu·K/mol)	2.672 ± 0.008	2.67 ± 0.01
*θ* (K)	−1.1 ± 0.7	−0.1 ± 0.3

**Table 4:** Compounds with Co-Co exchange interaction (spatial interaction dimension). Reference. Co coordination. Exchange path: carboxylate paths (Co-O-C-O-Co) { in parenthesis (#) number of carboxylate paths, type of path}, and oxygen mediated path (Co-O-Co). Co-Co distance. *C*, Curie constant (for comparison purposes, the *C* value given in this Table assumes 2 Co per f.u; for a Co(II) dimer, *C* = 3.75 emu·K/mol for spin only 3/2), *θ*, Curie–Weiss temperature, *g*, gyromagnetic factor (g* in effective spin *S* = 1/2 ground state), sub-indexes indicate directions (*x*, *y*, *z*), or spin subsystems. Exchange interaction Hamiltonian, transformed to the H_H_ = −2*J**S*****_1_*S*_2_** (Heisenberg), H_I_ = −2*JS_z1_S_z2_* (Ising) or H_AN_ = −2J(S_z1_S*_z2_* + e_x_S_x1_S_x2_ + e_y_S_y1_S*_y2_*) (Anisotropic) conventions, Spin, *S* = 3/2 model, or effective spin S* = 1/2 model. *J*/k_B_, exchange interaction constant. *D*/k_B_, anisotropy constant.

	Ref.	Coord.	Exch. Path	Co-Co (Å)	C (emu·K/mol dimer)	θ (K)	g	Model Spin	*J*/k_B_ (K)	*D*/k_B_ (K)
Co-Co Carboxylate Exchange Paths										
Co_2_(esp)_2_(EtOH)_2_ (dimer)	[55]	CoO4L L-outwards	(4) syn-syn	2.7245	∼4.4		g_⊥_ = 2.10 g_‖_ = 3.5	H_H_ 3/2	−9.49 AF	−79
**{[CoCxAPy]·2.15 H_2_O}_n_** **(2LL dimer)**	**This work.**	**CoN_2_O_4_** **apical**	**(2) syn-syn**	**3.9684**	**5.35**	**−1.1**	***g*_x_ = 2.40** ***g*_y_ = 2.58** ***g*_z_ = 1.96**	**H_H_** **3/2**	**−0.32**	**82** **η = 0.13**
**“**		**“**	**“**	**“**			g*_x_ = 4.07 g*_y_ = 5.57 g*_z_ = 1.94	**H_AN_** **1/2**	**−2.3** **e_x_ = 0.2** **e_y_ = 0.2**	
[Co(Htatb)(bimbp)]·DMF (dimer)	[54]	CoN_2_O_4_ apical	(2) syn-syn	4.156	3.73	−7.21	2.28	H_H_ 3/2	−6.65 AF	
[Co(Htatb)(1,3-bimyb)] (dimer)	[54]	CoN_2_O_4_ apical	(2) syn-syn	4.176	3.69	−8.44	2.4	H_H_ 3/2	−5.23 AF	
[{Co(phen)}_2_(fum)_2_] (dimer)	[58]	CoN_2_O_4_ adjacent	(2) distorted syn-syn	4.464	5.93		g*_‖_ = 6.0 g*_⊥_ = 3.5	H_AN_ 1/2	<1K Ferro	
dipy_2_Co_2_(μ-OOCCMe_3_)_2_ (OOCCMe_3_)_2_ (dimer)	[59]	CoN_2_O_4_ CoO_5_ adjacent	(2) syn-syn	4.383	5.688			H_H_ 3/2		
[Co(3,4-pyda)(H_2_O)_2_]_n_·nH_2_O (bilayer-dimer)	[23]	CoNO_5_ CoO_5_	(1) syn-anti	4.773	4.39	8.3		H_H_ 3/2		
[Co{OOC(CH_2_)_n-2_-COO}(H_2_O)_2_] d-12Co (chain)	[60,61]	CoO_6_	(1) anti-anti	5.93	6.0	−19.7		H_I_ 1/2	−1.02	
[Co{CH_3_(CH_2_)_n-2_COO}_2_(H_2_O)_2_] m-12Co, m-20Co (chain)	[61]	CoO_6_	(1) anti-anti	6.3	7.0	−17.4 −31.8		H_I_ 1/2	−0.65	
**Co-Co Carboxylate and Oxygen Mediated Exchange Paths**										
[Co_2_(H_2_O)_4_(Hbidc)_2_]_n_ (chain)	[62]	CoNO_5_ outwards	(2) syn-syn (2) Co-O-Co	3.114	5.35	1	2.39	H_H_ 3/2	1.21 Ferro	
{Co((κ_1_-κ_1_)-( κ_1_-μ_2_)-μ_4_TDC)(μ_2_H_2_O)0.5(H_2_O)}_n_ (dimer)	[63]	CoO_6_	(2) syn-syn (1) Co-O-Co	3.212	7.06	2.63	g* = 5.2	H_I_ 1/2		
bpyCo_2_(μ_2_-O,η_2_-OOCCMe_3_) (μ_2_-O,O’-OOCCMe_3_)_2_ (η_2_-OOCCMe_3_) (unsymmetrical dimer)	[64]	CoNO_5_ CoO_5_	(2) syn-syn (1) Co-O-Co	3.272					Ferro	
[Co_2_(μ-OAc)_3_(urea)(tmen)_2_]- [OTf] (dimer)	[56]	CoN_2_O_4_ adjacent	(2) syn-syn (1) Co-O-Co	3.4813	5.59		4.73 (dimer) (2 × 2.63)	H_H_ L = 1	26	57
[Co_2_(μOAc)2(μ-AA)(urea) (tmen)_2_][OTf] (3) (dimer)	[65,66]	CoN_2_O_4_ adjacent	(2) syn-syn (1) Co-O-Co	3.4316	6.48		g_‖_ = 3.0 g_⊥_ = 2.6 g_av_ = 2.66	H_H_ 3/2	−5.2	43
[Co_2_(μ-H_2_O)(μOAc)_2_(OAc)_2_ (tmen)_2_] (1) (dimer)	[65,66,67]	CoN_2_O_4_ adjacent	(2) syn-syn (1) Co-O-Co	3.597	6.42	−8.5	g_‖_ =2.9 g_⊥_ = 2.5 g_av_ =2.64	H_H_ 3/2	−1	64.3
[Co_2_(μ-OAc)_2_(OAc)_2_(μ-H_2_O) (phen)_2_] (dimer)	[68]	CoN_2_O_4_ adjacent		3.57	6.5					
[Co_3_(OH)_2_(3,4-pyda)_2_(H_2_O)_2_]_n_	[23]	CoNO_5_ CoO6	(1) syn-syn (1) Co-O-Co	2.988				H_H_ 3/2	−8.3	
Co_2_(H_2_O)(C_6_H_4_O_2_N)_4_·0.5CH_3_- CH_2_OH·0.5H_2_O (1) (dimer)	[22]	CoN_2_O_4_	(1) syn-syn (1) Co-O-Co		3.17	−31.2	2.53	H_H_ 3/2	−7.9	
Co_2_(H_2_O)(C_6_H_4_O_2_N)_4_·C_6_H_5_CH_2_OH (dimer)	[22]	CoN_2_O_4_	(1) syn-syn (1) Co-O-Co		3.42	−33.1	2.63	H_H_ 3/2	−8.3	
{[Co(dpyo)(tp)(H_2_O)_2_]· [Co(H_2_O)_6_]·(tp)·(H_2_O)}_n_ (chain)	[69]	CoO_6_	(1) syn-syn (1) Co-O-Co	3.742	6.56			H_I_ 1/2	−12.4	
**Co-Co Oxalate Exchange Path**										
Na_2_Co_2_(C_2_O_4_)_3_(H_2_O)_2_ (2 LL-dimer)	[57], [70,71,72]	CoO_6_	(2) oxalate Co-O-C-O-C	5.393	4.86	−36	*g*_x_* = 2.8 *g*_y_* = 3.3 *g*_z_* = 5.75	H_AN_ 1/2	−30.5 e_x_ = 0.31 e_y_ = 038	
[Co_2_(ox)tpmc](ClO_4_)2.3H_2_O (asymmetric dimer)	[73]	CoN_4_O_2_	(2) oxalate				g_a_ = 3.42 g_b_ = 4.02	H_H_ 3/2	−12.9	6.6
“	[74]	“	“				*g*_a_* = 4.43 *g*_b_* = 3.12	H_I_ 1/2	−16	

**Table 5:** Co-Co dimers with slow magnetic relaxation. Reference. Co coordination. Bridges (number) type. Co-Co distance. *J* exchange constant in *S* = 3/2 Heisenberg isotropic model (H_H_ = −2*J***S_1_**.**S_2_**). *D*- Uniaxial anisotropy constant. *H* – Field applied to observe relaxation. _o_ and *U*_eff_- Arrhenius law parameters for Orbach magnetic relaxation. n and C Raman process parameters.

Compound	Ref.	Coord.	Bridges	Co-Co (Å)	*J*/k_B_ (K)	*D*/k_B_ (K)	*H* (kOe)	*τ*_o_ (s)	*U*_eff_/k_B_ (K)	n	C/K^-n^ (s^−1^)
[(dmso)CoL^2^(μ-(m-NO_2_) C_6_H_4_COO)Co(NCS)] (2) symmetric	[75]	CoN_2_O_4_ CoNO_5_	(2)Co-O-Co (1)Nitrobenzoate	2.98(2)	6.52	11.7 −1.86	3	6.7 × 10^−13^	31.3		
[Co_2_(bedmpzp)_2_(μ-Cl)_2_] (PF_6_)_2_	[76]	CoN_4_Cl_2_	(2) Co-Cl-Co	4.0061	2.0	273	5	Yes No data	Yes No data		
[CoCl_2_LC^7^] (2)	[77]	CoN_3_Cl_2_	π-π contacts	5.662	1.03	219	2	1.07 × 10^−7^	14.6		
[CoCl_2_LC^14^] (5)	[77]	CoN_3_Cl_2_	π-π contacts	4.515	0.76	125	2	5.96 × 10^−8^	40.5		
[Co_2_(pypz)_2_(μ1,1-N_3_)_2_ (N_3_)_2_]·2CH_3_OH	[78]	CoN_6_	(2) Co-N-Co	3.3417	12.8	2.14	3	HF 5.15 × 10^−15^ LF 1.5	95	1.6	412
**{[CoCxAPy)]·2.15 H_2_O}_n_** **(2 LL-dimer)**	**This work**	**CoN_2_O_4_** **apical**	**(2) syn-syn** **carboxylate**	**3.9684**	−0.45	82 *E/D* = 0.13	5	HF 1.9 × 10^−6^ LF 2.0	6.7		

## Data Availability

Data supporting reported results can be provided by the corresponding author A.A. upon request.

## Supplementary Materials

Supplementary Information (SI). In section SI1, we provide additional crystal data and structure information. Figure S1: FTIR spectra for the compounds **1** and **2**. Figure S2: Thermogravimetric curves. Table S1: Main thermal decomposition steps of compounds **1** and **2**. Figure S3: EDX spectrum for compound **2**. Figure S4: XRF spectrum for compound **2**. Figure S5: DSC curves for compound **1**. Figure S6: PXRD diffractograms of the compounds **1** and **2** at room temperature. Table S2: Bond distances and angles for **1**. Figure S7: View of the asymmetric unit of coordination polymer **2**. Figure S8. View of the 2D MOF in the crystal structure of **2**. Table S3: Bond distances and selected angles for **2**. In the following sections (SI2, SI3 and SI4), we provide additional information about the magnetic properties of the studied compounds. Figure S9: Temperature dependence of the *χ*·*T* product for pure **1** and diluted compound **2** showing the high temperature fit to a Curie-Weiss law with parameters of Table 2 in the main text. Figure S10: Magnetic entropy per Co(II) ion as a function of temperature for different applied magnetic fields. Figure S11: In-phase component of the ac magnetic susceptibility as a function of frequency. Figure S12: In-phase amplitude of the low frequency process at *T* = 2K as a function of the applied dc magnetic field.
