# Supplementary material for: Slow Magnetic Relaxation in {[CoCxAPy)] 2.15 H2O}n MOF Built from Ladder-Structured 2D Layers with Dimeric SMM Rungs
Source: Molecules. 2021 Sep 16;26(18):5626. doi: 10.3390/molecules26185626 (PMC8466197; doi:10.3390/molecules26185626)
Supplement: Supplementary file 1 [file molecules-26-05626-s001.zip › molecules-1287606-supplementary.pdf]

Supplementary Information

Slow magnetic relaxation in {[CoCxAPy)] 2.15 H_2O}$_n$ MOF built from ladder-structured 2D layers with dimeric SMM rungs

Ana Arauzo [1,*], Elena Bartolomé [2], Javier Luzón [1,3], Pablo J. Alonso [1], Angelica Vlad[4] , Maria Cazacu [4], Mirela F. Zaltariov [4], Sergiu Shova [4], Juan Bartolomé [1], and Constantin Turta [4,5,†]

[1] Instituto de Nanociencia y Materiales de Aragón (INMA), CSIC-Universidad de Zaragoza, Pedro Cerbuna 12, 50009 Zaragoza, Spain.; jluzon@unizar.es (J.L.), alonso@unizar.es (P.J.A.); barto@unizar.es (J.B.)
[2] Department of Mechanical Engineering. Escola Universitària Salesiana de Sarrià (EUSS). Passeig de Sant Joan Bosco, 74, 08017, Barcelona, Spain; ebartolome@euss.es (E.B.);
[3] Centro Universitario de la Defensa, Ctra. de Huesca s/n, E-50090 Zaragoza, Spain; jluzon@unizar.es (J.L.);
[4] Department of Inorganic Polymers, "Petru Poni" Institute of Macromolecular Chemistry, Aleea Gr. Ghica Voda 41A, 700487 Iasi, Romania; avlad@icmpp.ro (A.V.); mcazacu@icmpp.ro (M.C.); zaltariov.mirela@icmpp.ro (M.F.Z.); shova@icmpp.ro (S.S.)
[5] Institute of Chemistry, Academy of Sciences of Moldova, Academiei 3, MD-2028 Chisinau, Moldova (C.T.);
† Constantin Turta passed away on 23th March 2015.
* Correspondence: aarauzo@unizar.es;

- **SI1. Crystal Data and Structure** **p.2**
- **SI2. Magnetic Susceptibility** **p.11**
- **SI3. Magnetic Entropy** **p.11**
- **SI4. Ac Susceptibility Measurements** **p.12**

SI1.Crystal Data and Structure

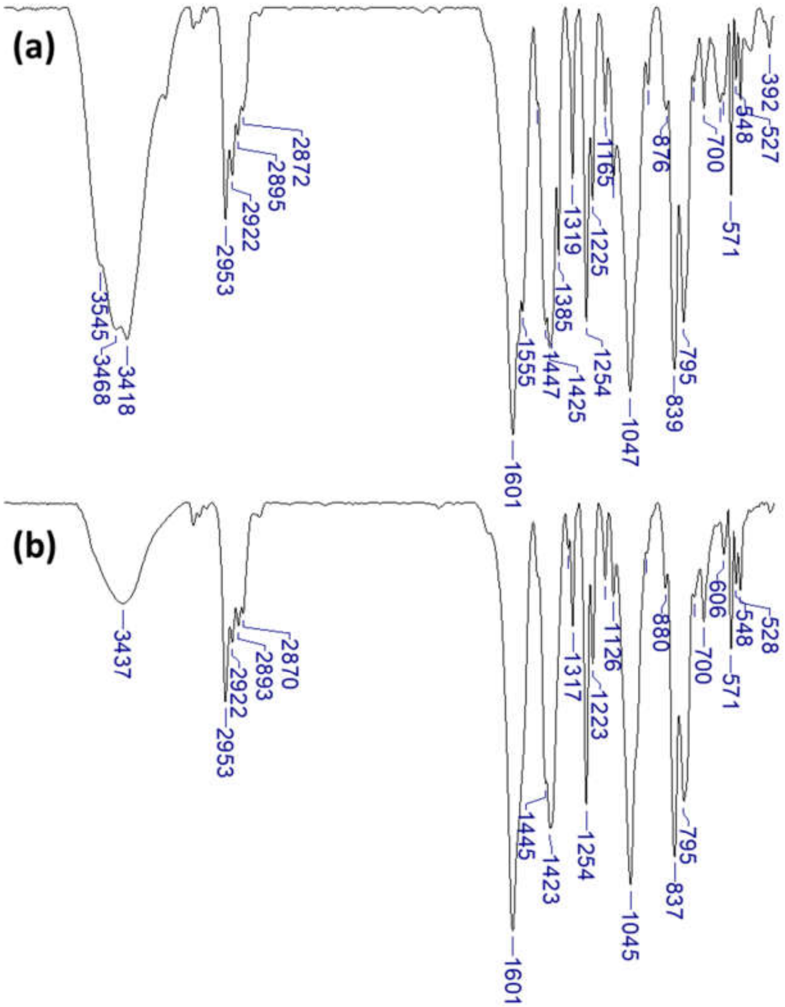

Figure S1*. FTIR spectra for the compounds* ***1*** *(a) and* ***2*** *(b).*

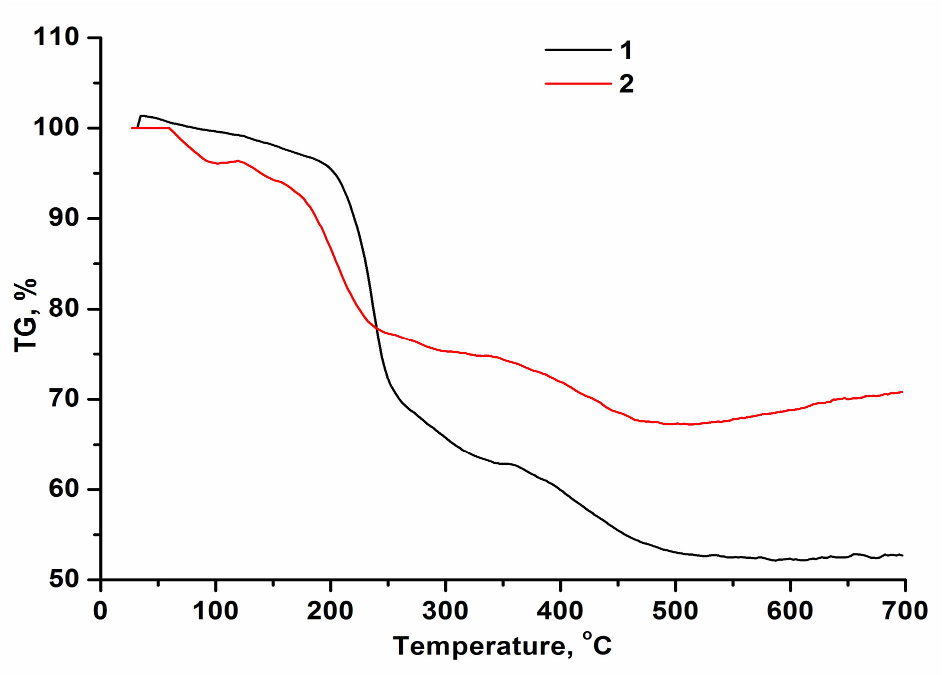

Figure S2. *Thermogravimetric curves for the compounds* ***1*** *and* ***2****.*

Table S1. The main thermal decomposition steps of compounds 1 and 2 in the inert atmospher as emphasized by thermogravimetric analysis.

Compound	Decomposition stage	Temperature range, °C	T_{max}^{a},°C	Δw^{b}, %
**1**	I	40.7 - 86.6	55.8	1.75
	II	124.0-197.9	130.5	3.37
	III	197.9-249.2	237.5	24.41
	IV	249.2-343.4	296.2	8.63
	V	362.1-475.9	403.4	9.96
**2**	I	41.0 - 94.2	60.5	5.35
	II	121.2-159.6	139.1	2.68
	III	175.8-228.2	204.2	14.77
	IV	259.2-294.1	276.4	2.19
	V	346.1-457.7	437.2	4.07

[a] T_{max}- temperature corresponding to the maximum rate of decomposition;
[b] Δw-weight loss percentage corresponding to the degradation stage.

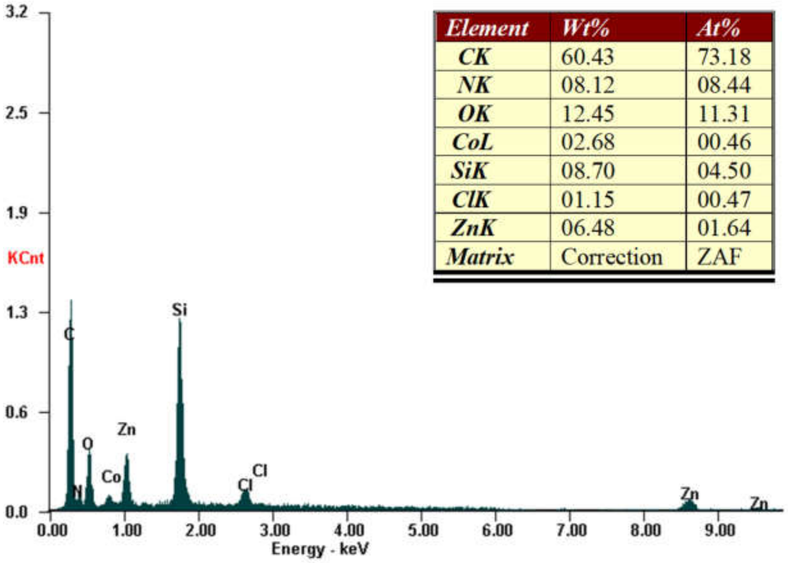

Element	Wt%	At%
CK	60.43	73.18
NK	08.12	08.44
OK	12.45	11.31
CoL	02.68	00.46
SiK	08.70	04.50
ClK	01.15	00.47
ZnK	06.48	01.64
Matrix	Correction	ZAF

Figure S3. *EDX spectrum for the compound **2**, revealing a Co:Zn molar ratio of 0.22:0.78.*

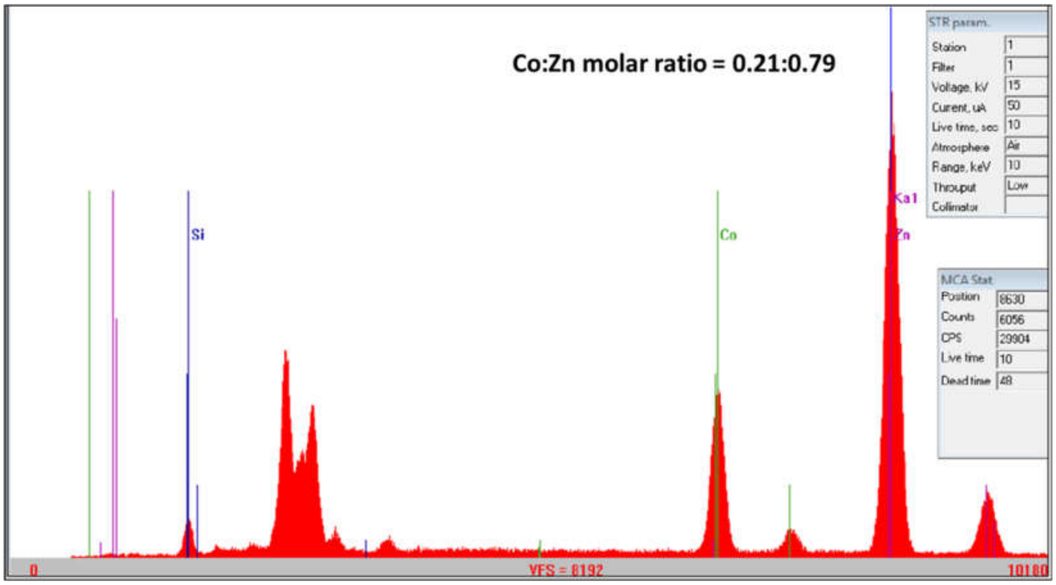

Figure S4. *XRF spectrum for the compound **2**.*

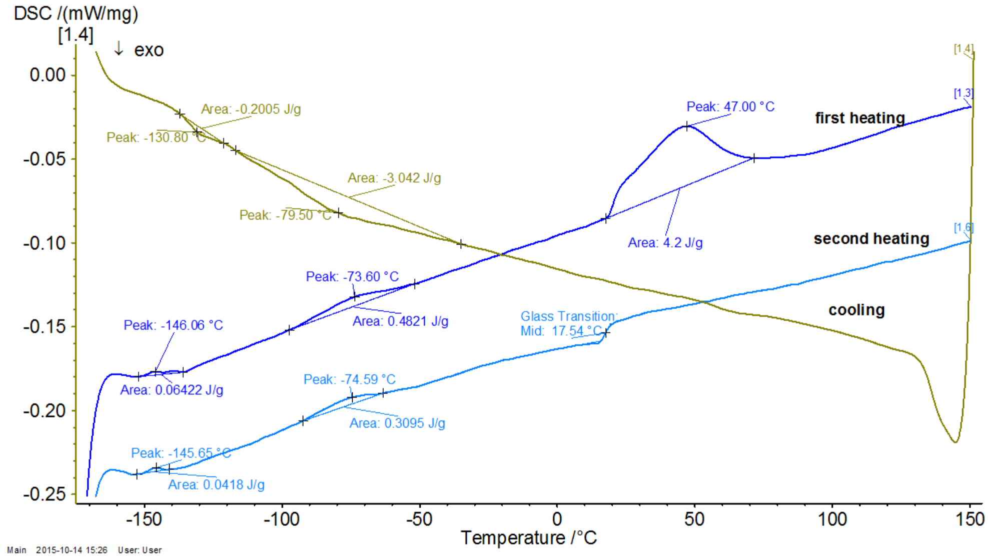

***Figure S5**. The DSC curves illustratively shown for compound **1**.*

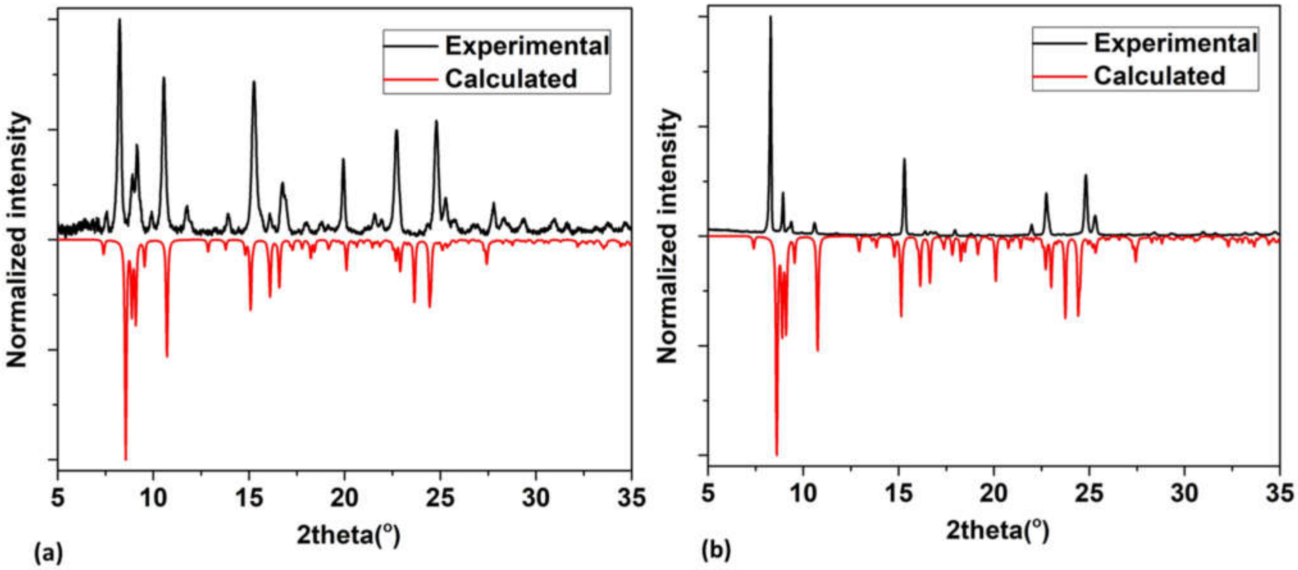

***Figure S6**. PXRD diffractograms of the compounds **1** (a) and **2** (b) recorded at room temperature as compared with those simulated on the basis of single crystal X-ray diffraction experiments.*

Table S2*. Bond distances (Å) and selected angles (°) for compound* ***1****.*

C1-C2	1.521(9)	C15-C16	1.378(9)
C1-O1	1.262(6)	C15-N2	1.447(7)
C1-O2	1.243(6)	C16-C17	1.378(7)
C2-C3	1.544(8)	C17-N1	1.316(7)
C3-C4	1.515(8)	C18-C19	1.387(8)
C4-Si1	1.855(6)	C18-C22	1.384(9)
C5-Si1	1.842(7)	C18-N3	1.431(7)
C6-Si1	1.845(8)	C19-C20	1.383(7)
C7-Si2	1.856(8)	C20-N4	1.335(7)
C8-Si2	1.863(7)	C21-C22	1.382(7)
C9-C10	1.527(9)	C21-N4	1.352(7)
C9-Si2	1.857(7)	Co1-N1	2.162(4)
C10-C11	1.527(10)	Co1-N4[2]	2.164(4)
C11-C12	1.504(9)	Co1-O1	2.021(4)
C12-Co1[1]	2.512(6)	Co1-O2[3]	2.020(4)
C12-O4	1.261(8)	Co1-O4[4]	2.213(4)
C12-O5	1.255(8)	Co1-O5[4]	2.164(4)
C13-C14	1.383(8)	N2-N3	1.238(7)
C13-N1	1.345(8)	O3-Si1	1.635(5)
C14-C15	1.378(8)	O3-Si2	1.633(5)

O1-C1-C2	115.3(6)	O1-Co1-O52	151.48(19)
O2-C1-C2	120.0(5)	O24-Co1-N1	87.65(17)
O2-C1-O1	124.7(6)	O24-Co1-N43	94.88(17)
C1-C2-C3	115.5(6)	O24-Co1-O1	118.41(18)
C4-C3-C2	114.6(6)	O24-Co1-O42	149.50(18)
C3-C4-Si1	115.0(5)	O24-Co1-O52	89.80(19)
C10-C9-Si2	114.3(5)	O52-Co1-N43	91.09(16)
C9-C10-C11	112.9(6)	O52-Co1-O42	60.08(18)

C12-C11-C10	113.9(6)	C17-N1-C13	117.5(5)
O4-C12-C11	120.1(7)	C17-N1-Co1	120.2(4)
O5-C12-C11	118.8(7)	N3-N2-C15	111.8(5)
O5-C12-O4	121.1(6)	N2-N3-C18	113.9(5)
N1-C13-C14	122.1(6)	C20-N4-C21	118.0(5)
C15-C14-C13	119.1(6)	C20-N4-Co1[5]	122.7(4)
C14-C15-N2	116.0(5)	C21-N4-Co1[5]	119.3(4)
C16-C15-C14	118.9(5)	C1-O1-Co1	132.0(4)
C16-C15-N2	125.1(5)	C1-O2-Co1[4]	145.5(4)
C15-C16-C17	117.9(5)	Si2-O3-Si1	153.6(3)
N1-C17-C16	124.4(6)	C12-O4-Co1[1]	88.2(4)
C19-C18-N3	115.5(5)	C12-O5-Co1[1]	90.6(4)
C22-C18-C19	118.8(5)	C5-Si1-C4	110.1(3)
C22-C18-N3	125.7(5)	C5-Si1-C6	109.3(4)
C20-C19-C18	118.1(6)	C6-Si1-C4	111.7(3)
N4-C20-C19	123.6(6)	O3-Si1-C4	108.1(3)
N4-C21-C22	121.8(6)	O3-Si1-C5	109.0(3)
C21-C22-C18	119.6(6)	O3-Si1-C6	108.6(3)
N1-Co1-N43	177.47(19)	C7-Si2-C8	108.8(4)
N1-Co1-O42	87.24(17)	C7-Si2-C9	110.0(4)
N1-Co1-O52	89.08(16)	C9-Si2-C8	111.3(4)
N43-Co1-O4[2]	90.66(17)	O3-Si2-C7	110.4(3)
O1-Co1-N1	88.10(16)	O3-Si2-C8	109.1(3)
O1-Co1-O4[2]	91.43(18)	O3-Si2-C9	107.3(3)

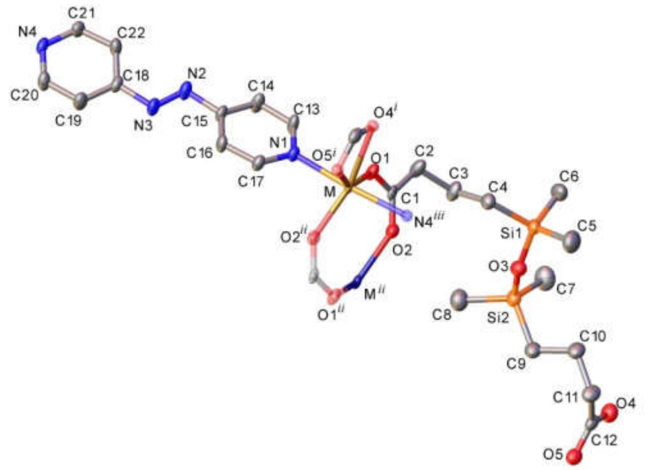

Figure S7. *View of the asymmetric unit of coordination polymer* **$\{[Zn_{0.82}Co_{0.18}CxAPy)]\cdot1.5\ CH_3OH\}_n$ *(2)*** *with atom labelling and thermal ellipsoids at 50% level. H atoms are omitted for clarity. Atoms obtained by symmetry-transformations are displayed semi-transparent. Symmetry codes: $^{i)}$ x, y – 1, z – 1; $^{i)}$ 1 - x, 1 - y, 1 - z; $^{i)}$ x, y, 1 + z.*

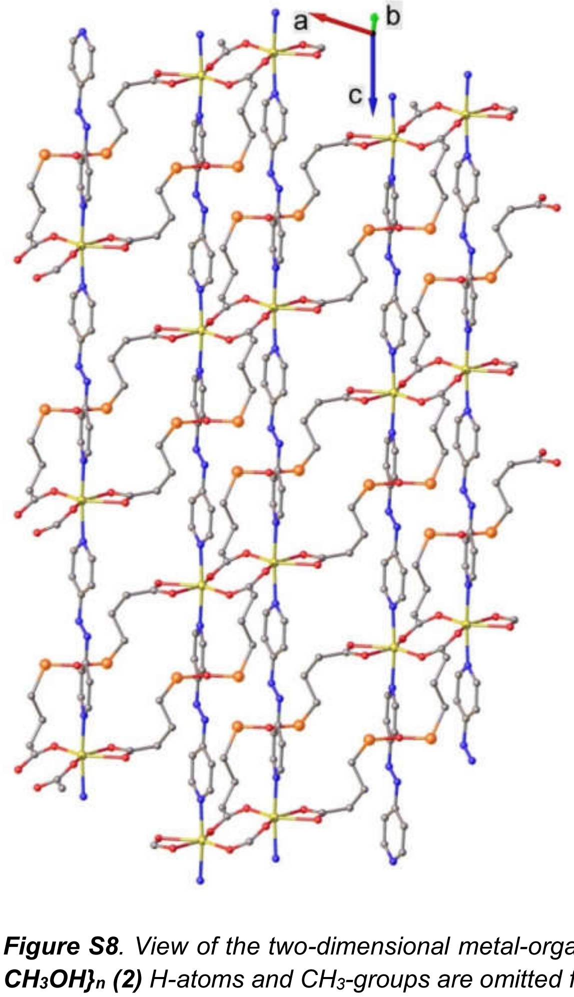

Figure S8. *View of the two-dimensional metal-organic framework in the crystal structure of* **$\{[Zn_{0.80}Co_{0.20}CxAPy)]\cdot1.5\ CH_3OH\}_n$ *(2)*** *H-atoms and CH_3-groups are omitted for clarity. Symmetry codes: ^{1}2 - x, 1 - y, 1 - z; ^{2}x, y, z - 1;^{3}1 - x, 1 - y, -z; ^{4} x, y, 1 + z.*

***Table S3**. Bond distances (Å) and selected angles (°) for **2**.*

M-O1	2.022(2)	N2-C15	1.433(4)
M-O2[1]	2.037(2)	N3-C18	1.435(4)
M-O4[2]	2.295(2)	N4-C20	1.331(4)
M-O5[2]	2.145(2)	N4-C21	1.342(4)
M-N1	2.172(2)	C1-C2	1.513(4)
M-N4[3]	2.167(2)	C2-C3	1.534(4)
Si1-O3	1.634(2)	C3-C4	1.525(5)
Si1-C4	1.870(3)	C9-C10	1.523(5)
Si1-C5	1.856(4)	C10-C11	1.535(5)
Si1-C6	1.854(4)	C11-C12	1.509(4)
Si2-O3	1.634(2)	C13-C14	1.384(4)
Si2-C7	1.855(4)	C14-C15	1.379(4)
Si2-C8	1.851(4)	C15-C16	1.379(4)
Si2-C9	1.861(4)	C16-C17	1.381(4)
O1-C1	1.257(3)	C18-C19	1.376(5)
O2-C1	1.248(4)	C18-C22	1.383(5)
O4-C12	1.249(4)	C19-C20	1.380(4)
O5-C12	1.252(4)	C21-C22	1.378(4)
N1-C13	1.337(4)	O6-C23	1.373(7)
N1-C17	1.338(4)	O7-C24	1.352(8)
N2-N3	1.233(4)		

O1-M-O2[1]	121.03(9)	C17-N1-M	119.0(2)
O1-M-O4[2]	90.93(9)	C17-N1-Co1	119.0(2)
O1-M-O5[2]	149.06(10)	N3-N2-C15	113.1(3)
O1-M-N1	87.59(9)	N2-N3-C18	114.4(3)
O1-M-N4[3]	90.81(9)	C20-N4-M[5]	122.5(2)
O2[1]-M-O4[2]	147.38(9)	C20-N4-C21	117.7(3)

O21-M-O5^{2}	89.39(9)	C21-N4-M^{5}	119.7(2)
O2^{1}-M-N1	87.71(9)	O1-C1-C2	116.0(3)
O2^{1}-M-N4^{3}	96.01(9)	O2-C1-O1	125.5(3)
O5^{2}-M-O4^{2}	58.25(9)	O2-C1-C2	118.5(3)
O5^{2}-M-N1	88.24(9)	C1-C2-C3	116.4(3)
O5^{2}-M-N4^{3}	91.41(9)	C4-C3-C2	113.4(3)
N1-M-O4^{2}	87.30(9)	C3-C4-Si1	114.5(2)
N4^{3}-M-O4^{2}	89.34(9)	C10-C9-Si2	114.4(3)
N4^{3}-M-N1	176.26(10)	C9-C10-C11	113.4(3)
O3-Si1-C4	108.40(14)	C12-C11-C10	112.2(3)
O3-Si1-C5	108.42(17)	O4-C12-O5	119.9(3)
O3-Si1-C6	109.30(17)	O4-C12-C11	120.6(3)
C5-Si1-C4	110.57(18)	O5-C12-C11	119.5(3)
C6-Si1-C4	110.88(17)	N1-C13-C14	122.5(3)
C6-Si1-C5	109.2(2)	C15-C14-C13	118.9(3)
O3-Si2-C7	110.50(17)	C14-C15-N2	117.0(3)
O3-Si2-C8	109.06(15)	C16-C15-N2	123.9(3)
O3-Si2-C9	107.45(15)	C16-C15-C14	119.0(3)
C7-Si2-C9	109.48(19)	C15-C16-C17	118.5(3)
C8-Si2-C7	109.15(19)	N1-C17-C16	123.1(3)
C8-Si2-C9	111.19(19)	C19-C18-N3	115.7(3)
C1-O1-M	131.0(2)	C19-C18-C22	118.8(3)
C1-O1-Co1	131.0(2)	C22-C18-N3	125.5(3)
C1-O2-M1	143.5(2)	C18-C19-C20	119.2(3)
Si2-O3-Si1	153.51(16)	N4-C20-C19	122.7(3)
C13-N1-M	123.1(2)	N4-C21-C22	123.2(3)
C13-N1-C17	117.8(3)	C21-C22-C18	118.4(3)
C13-N1-Co1	123.1(2)		

Symmetry codes: ^{1}1-X,2-Y,1-Z; ^{2}+X,-1+Y,-1+Z; ^{3}+X,+Y,1+Z; ^{4}+X,1+Y,1+Z; ^{5}+X,+Y,-1+Z

SI2. Magnetic Susceptibility

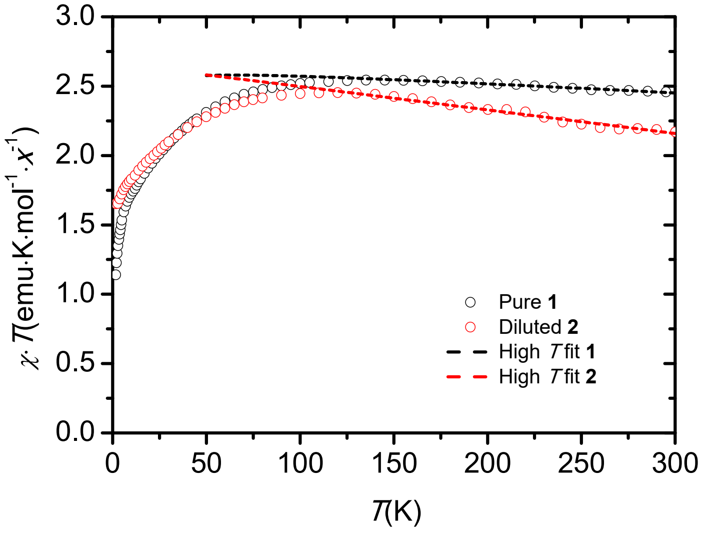

Figure S9. *Temperature dependence of χT for pure **1** (x=1) and diluted compound **2** (x=0.2), measured at H=1 kOe. Dotted lines show the high temperature fit to a Curie-Weiss law with parameters of Table 3 in the main text.*

SI3. Magnetic Entropy

When magnetic entropy is analyzed, we observe that entropy change below 10 K reaches values accounting 0.5-0.6 R per Co ion for the different magnetic fields. This amount is indicating that the exchange coupling in the dimmer is not Ising type which would produce two split doublets with change of entropy computing only 0.34 R. On the other hand, an isotropic ferromagnetic interaction FM would give much lower contribution.

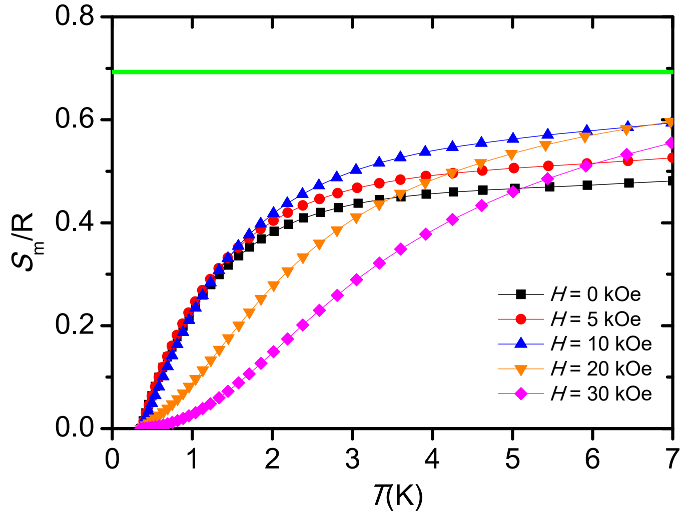

Figure S10. *Magnetic entropy per Co(II) ion as a function of temperature for different applied magnetic fields. The threshold Rln2 = 0.693 R is shown as a reference (compound **1**).*

SI4. Ac Magnetic Susceptibility Measurements

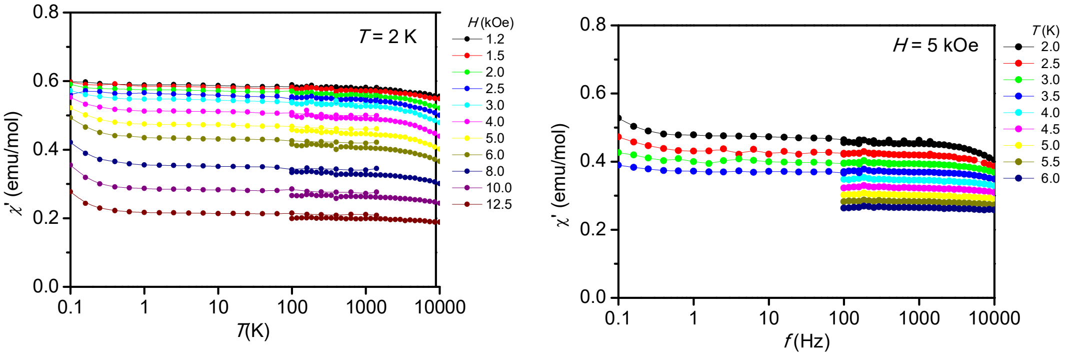

***Figure S11**. In-phase component of the ac magnetic susceptibility as a function of frequency. Left: T = 2 K for various dc applied fields. Right: H = 5 KOe for various temperatures (compound **1**).*

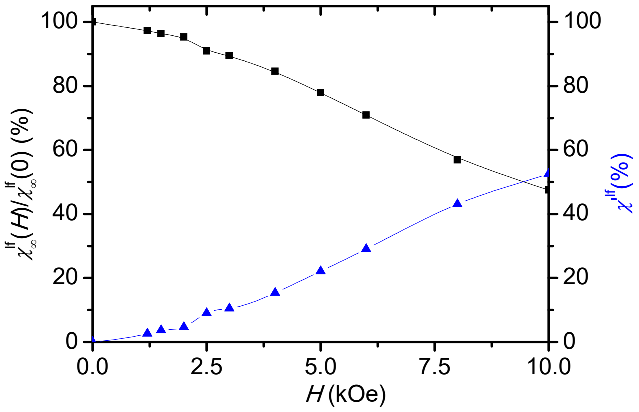

***Figure S12**. In-phase amplitude of the low frequency process at T=2 K as a function of the applied dc magnetic field (blue triangles). The value is calculated as the maximum value, χ'(100 Hz,H=0), minus the χ'(100 Hz,H) and plotted in percentage. The left axis displays the remaining χ' after blocking (compound **1**).*

In the low frequency window, below 1 Hz, the decrease in χ' for increasing magnetic field in conjuction with the pressence of a raising low frequency process, $\chi'' > 0$ is a sign of a dominant low frequency relaxation process which is already blocked at 10 Hz. Figure S12 displays graphically the decrease of χ' at 100 Hz and 2K as a function of field, in percentage. That percentage corresponds to the contribution to the susceptibility of the centers which are not participating in this low frequency process, in other words, the low frequency process associated adiabatic susceptibility, χ_∞^{lf} .

As for the high frequency process, if we assume that beyond 10 kHz χ_∞ =0, the obtained $\tau_{hf}(H)$ values are not coincident with the relaxation times determined with the position of the maxima in χ'' curves (data shown in Figure 7a) and the *H* dependence gives a tendency contrary to observation. This apparent contradiction is explained as due to a non-zero adiabatic susceptibility at higher frequencies above 10 kHz, ascribed to spins relaxing through much faster mechanisms. In order to determine this contribution, the value τ_{hf} (2K,5kOe) has been used as a reference to find χ_∞ for the other temperature values. We have considered that χ_∞ has the same percentage for the whole set of temperatures, namely $\chi_\infty(T) = 0.775\cdot\chi'(T,100\text{Hz}) = 0.775\cdot\chi_\infty^{lf}$. Therefore for a magnetic field of 5 kOe and a temperature of 2 K, about 22 % of the centers relax at very low frequency, 18% in the high frequency process, and the remaining 60 % are in equilibrium, with very fast relaxation times, out of the experimental frequency window. The so obtained $\tau_{hf}(T)$ are depicted in Fig. 7d. In order to corroborate the procedure, the relaxation time values are also obtained taking a null adiabatic contribution. The variation with *T* in the later are similar, although the values are much lower and do not match experimental curves.
